# A multimethod computational simulation approach for investigating mitochondrial dynamics and dysfunction in degenerative aging

**DOI:** 10.1111/acel.12644

**Published:** 2017-08-16

**Authors:** Timothy E. Hoffman, Katherine J. Barnett, Lyle Wallis, William H. Hanneman

**Affiliations:** ^1^ Center for Environmental Medicine College of Veterinary Medicine and Biomedical Sciences Colorado State University Fort Collins CO 80523 USA

**Keywords:** agent‐based modeling, computational biology, deterministic modeling, mitochondrial dysfunction, mitophagy, oxidative stress

## Abstract

Research in biogerontology has largely focused on the complex relationship between mitochondrial dysfunction and biological aging. In particular, the mitochondrial free radical theory of aging (MFRTA) has been well accepted. However, this theory has been challenged by recent studies showing minimal increases in reactive oxygen species (ROS) as not entirely deleterious in nature, and even beneficial under the appropriate cellular circumstances. To assess these significant and nonintuitive observations in the context of a functional system, we have taken an *in silico* approach to expand the focus of the MFRTA by including other key mitochondrial stress response pathways, as they have been observed in the nematode *Caenorhabditis elegans*. These include the mitochondrial unfolded protein response (UPR ^mt^), mitochondrial biogenesis and autophagy dynamics, the relevant DAF‐16 and SKN‐1 axes, and NAD ^+^‐dependent deacetylase activities. To integrate these pathways, we have developed a multilevel hybrid‐modeling paradigm, containing agent‐based elements among stochastic system‐dynamics environments of logically derived ordinary differential equations, to simulate aging mitochondrial phenotypes within a population of energetically demanding cells. The simulation experiments resulted in accurate predictions of physiological parameters over time that accompany normal aging, such as the declines in both NAD ^+^ and ATP and an increase in ROS. Additionally, the *in silico* system was virtually perturbed using a variety of pharmacological (e.g., rapamycin, pterostilbene, paraquat) and genetic (e.g., *skn‐1*,* daf‐16, sod‐2*) schemes to quantitate the temporal alterations of specific mechanistic targets, supporting insights into molecular determinants of aging as well as cytoprotective agents that may improve neurological or muscular healthspan.

## Introduction

Loss of mitochondrial function is one of the most well‐established characteristics of aging cells. Because of this, many researchers speculate that the decline in mitochondrial function is not only a complex consequence of biological aging, but may provide the earliest trigger in time‐dependent cellular senescence and degeneration (Van Raamsdonk & Hekimi, 2010; Wang & Hekimi, 2015). The first viable theory explaining this relationship is the mitochondrial free radical theory of aging (MFRTA), which suggests that increasing levels of mitochondrial ROS (mtROS) produced by cellular respiration and the electron transport chain (ETC) are responsible for age‐dependent damage, especially toward copies of mitochondrial DNA (mtDNA) (Harman, 1956; Balaban *et al*., 2005). According to this theory, when mtDNA and matrix proteins exist in an oxidatively damaged state, ETC function may be reduced and proteostasis may be lost, resulting in a deleterious cyclic propagation of mtROS generation and a decrease in cellular respiratory function. The MFRTA has been adapted and strengthened over the years to address many of the complexities and nuances associated with mitochondrial dysfunction in aging cells (Hekimi *et al*., 2011; Ziegler *et al*., 2015). For example, more recent findings suggest that the accumulation of loss‐of‐function mutations is not entirely mtROS‐dependent (Kujoth *et al*., 2005; Kennedy *et al*., 2013), and have demonstrated that minimal increases in endogenous mtROS may be fairly benign or, controversially, cytoprotective in nature (Yang & Hekimi, 2010; Gruber *et al*., 2011). These contrasting data warrant further investigation of this theory as a pertinent and highly multifaceted system in aging organisms.

One additional dimension that adds much value to the MFRTA involves the general response pathway by which the mitochondrion adapts to stress: the mitochondrial unfolded protein response (UPR^mt^). This response is conserved across an array of species, and presumably all eukaryotes, and may largely account for the interplay that dulls the downstream oxidative effects of mitochondrial damage during aging (Schulz & Haynes, 2015). The UPR^mt^ is activated directly or indirectly by a variety of stress signals originating from the ETC and the intramitochondrial environment (Yoneda *et al*., 2004; Nargund *et al*., 2012; Pulliam *et al*., 2014). This activated response has been extensively studied within the nematode *Caenorhabditis elegans*, where the UPR^mt^ is mediated by nuclear import of an important protein known as activating transcription factor associated with stress‐1 (ATFS‐1) (Nargund *et al*., 2012). This transcription factor is regulated in such a way that allows the cell to globally monitor mitochondrial stress and damage, and it effectively produces a widely protective transcriptional response when present in the nucleus. Among the several hundred transcripts upregulated by nuclear ATFS‐1 accumulation are those encoding for important mitochondrial proteins necessary for antioxidant defense and proteostasis, for example, *sod‐2/3*,* hsp‐60*,* hsp‐70*,* ymel‐1*, and *timm‐23* (Nargund *et al*., 2012; Mouchiroud *et al*., 2013). Additionally, this response negatively regulates the nuclear and mitochondrial expression of oxidative phosphorylation (OXPHOS) subunits to avoid the accumulation of superfluous proteins when mitochondria are already experiencing severe metabolic stress (Nargund *et al*., 2015). All of these functional changes promote mitochondrial health, which may in turn allow cells to live longer at a heightened state of performance.

Although widely beneficial for proteostasis and respiratory maintenance within cells, the UPR^mt^ has also been noted as a process that can sustain damage within the mitochondrial genome (Lin *et al*., 2016). It has been suggested that the UPR^mt^ can be overprotective by sustaining the numbers of both functional and severely defective mitochondria within a given cell, although the mechanism by which this occurs is far from clear. The activation of this stress response may allow for high respiratory function for the majority of a lifespan, but once enough mtDNA copies hold deletions or mutations within their sequences, it is possible that the balance may shift toward a deleterious cellular mechanism caused in part by sustained UPR^mt^ activation. This complete relationship has yet to be fully investigated.

Mitochondrial autophagy, or mitophagy, is another process that appears to play a critical role in biological aging. Poor regulation of this process allows for the accumulation of aberrant mitochondrial content, which has been postulated as a driving force for deleterious senescence states, particularly for age‐associated neurodegenerative diseases such as dementia, Alzheimer's disease (AD), and Parkinson's disease (PD) (Palikaras & Tavernarakis, 2012; Menzies *et al*., 2015). Drastic manipulation of mitochondrial biogenesis and mitophagy flux in *C. elegans* stress models has been shown to cause decreases in ATP production, increased oxidative burdens, and ultimately shortened lifespans (Palikaras *et al*., 2015), demonstrating how important it is for these processes to be tightly regulated to allow for healthy aging. These regulatory dynamics are also well conserved across a variety of animal species.

The causal role of dysfunctional mitochondrial dynamics in the process of biological aging remains fairly nebulous. All the aforementioned mitochondrial processes have been hypothesized as important factors that determine the rate and results of biological aging, yet it has been rather difficult to investigate all of these processes simultaneously and to quantitate their ultimate effects when compounded. Computational models have been able to provide an additional means to investigate such complex systems and have gained a great deal of interest specifically for the field of biogerontology (Mooney *et al*., 2016). The aim of using such an approach is to not only gain insights about the molecular determinants of tissue aging but to also gain quantitative insights about how such determinants can be targeted therapeutically.

**Figure 1:**
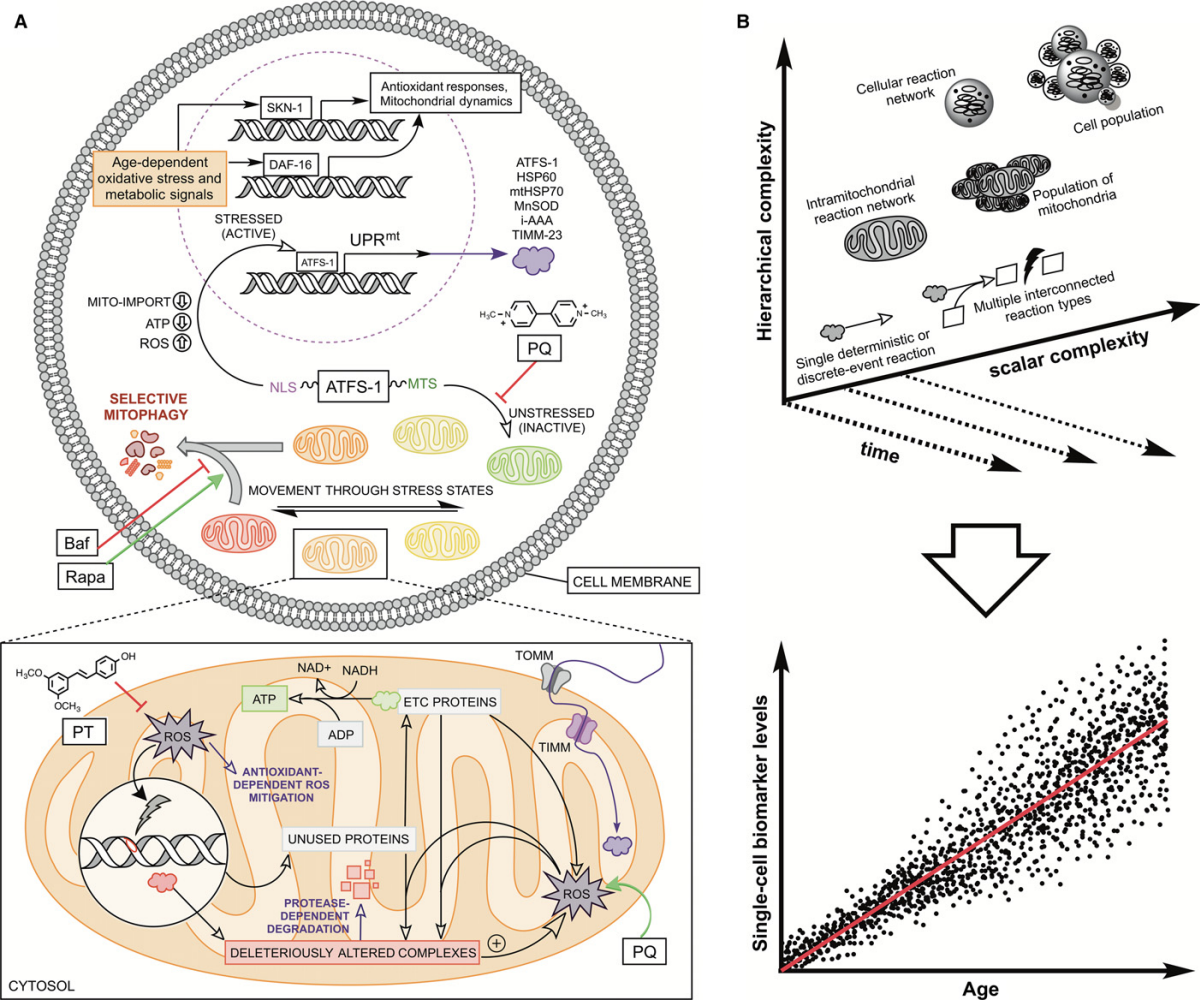
The integrated aging mechanism of interest and computational simulation approach. (A) Respiratory machinery produces both ATP and superoxide, which elicits different types of intramitochondrial damage (noted above as changes in mitochondrial stress states) and activation of various cellular stress response pathways. Such pathways focused on here are the UPR ^mt^, dictated by nuclear accumulation of transcription factor ATFS‐1, mitochondrial biogenesis and selective mitophagy, and activation of transcription factors DAF‐16 and SKN‐1. This integrated mechanism was also virtually probed by pharmacological agents that modulate either mitophagy, oxidative metabolism, or antioxidant defenses. Rapamycin induces mitophagy, bafilomycin hinders mitophagy, paraquat causes additional superoxide production and can activate the UPR ^mt^, and pterostilbene directly and indirectly boosts cellular antioxidant capacity. UPR ^mt^, mitochondrial unfolded protein response; NLS, nuclear localization signal; MTS, mitochondria targeting sequence; Rapa, rapamycin; Baf, bafilomycin; PQ, paraquat; PT, pterostilbene. (B) The simulation approach has been characterized as having two dimensions of complexity integrated over time: hierarchical or leveled complexity, and scalar complexity (i.e., magnitude of segments). These constructs were employed to produce large scale predictive datasets over time on the single‐cell level (denoted by black dots) which were distilled down into tissue‐level averages (denoted by red line).

Here we emphasize that the degenerative aging response is not a result of a single concrete cellular pathway, but is dependent upon specific magnitudes of multiple key mitochondrial pathways that allow for cellular senescence states and pathological phenotypes. To illuminate these theories in a more nuanced manner, we took an approach that highlights existing data and integrates them into a systemic computational modeling approach. Our theoretical model addresses vital cellular components and time‐dependent macromolecule quantities that account for biological aging within the model organism *C. elegans –* including bioenergetic functions of the mitochondrion, fundamental observations of the MFRTA, known elements of the UPR^mt^, and mitophagy‐dependent quality control processes (Fig. 1A). This framework was modeled using multimethod simulation software, allowing us to employ system‐dynamics elements (e.g., stocks and flows) as well as agent‐based functions (i.e., those that define discrete events, stochasticity, and seemingly autonomous cellular behavior). Additionally, our *in silico* approach included a hierarchical modeling scheme to control mitochondrial‐level dynamics within a population of energetically demanding cells, allowing us to generate predictive and probabilistic data at the single‐cell level (Fig. 1B). This theoretical model aims to provide a better understanding of all the significant cellular components that may account for mitochondrial function and dysfunction in biological aging with a quantitative backing that may aid in making experimental predictions for age‐related diseases, toxicological endpoints, and potential pharmacological or dietary anti‐aging therapies.

## Results

### Multimethod simulation development

The pathways of interest for the model were selected based on their broadly protective or damaging mitochondrial effects and their conserved significance toward the aging process observed over recent years. Such pathways were then compiled into a fully integrated system of mitochondrial health (Fig. 1A). This network was constructed within our simulation interface predominately by using deterministic frameworks to model both intramitochondrial reactions (Fig. S1A) and cytosolic and nuclear activities (Fig. S2A). To extend our investigation beyond traditional single‐method deterministic modeling, we overlaid an agent‐based framework for multiple events that have classically been difficult to model with differential equations and flow rates. This framework was primarily added to observe the aging kinetics of mitochondrial stress‐state phenotyping (Fig. S1B) and mito‐compromised cell states that eventually result in apoptotic cell death (Fig. S2B). Such agent‐based events occur discretely within the model and are bound by conditions or stochastic damage‐dependent frequencies. It is important to note that this multimethod framework was built expansively and hierarchically (Fig. 1B), where the model simulated a population of cells each with its own functioning reaction network and with its own population of mitochondria that were constantly undergoing biogenesis, stress‐dependent mitophagy, and mitochondrial recycling. Each mitochondrion within a cell's population also contained its own stochastic reaction network that controlled not only its own phenotypic stress state, but the state of the cell and thus the states of other mitochondria.

To fill this network with realistic parameters and relevant reaction schemes for the aging nematode, we gathered a vast set of information from the literature to determine: mitochondria counts and mtDNA counts (Table S1 and Box S1); mitochondrion‐level system‐dynamics and discrete‐event computational methods for oxidative metabolism, proteostasis, and heteroplasmic mtDNA analysis (Table S2 and Box S2); damage‐dependent agent‐based progression and recovery through mitochondrial stress states that lead to mitophagy (Box S3); cell‐level system‐dynamics computational methods surrounding the UPR^mt^ and mitochondrial protein import (Table S3 and Box S4); and agent‐based principles that govern stress‐dependent cellular degeneration (Box S5). These modeled reaction schemes consist of logically derived ordinary differential equations (ODEs) or discrete‐event statements, which simulate reactions such as ATP production, superoxide production, peroxide formation, mtROS elimination, baseline protein production and transcriptionally activated protein synthesis, protein import, and damage to and clonal expansion of mtDNA copies. The parameters used to fill these reaction schemes were (i) directly obtained and/or converted from previous models, (ii) assumed based on qualitative evidence, or (iii) individually optimized over 5000 parameter iterations to reach appropriate curves for the separate model components. All values and corresponding sources are reported as such in the Supporting Information.

We have drawn upon previous deterministic computational models of aging, including a deterministic mathematical analysis of superoxide production and disposition to complement *C. elegans* data (Gruber *et al*., 2011) and a compartmentalized dynamic model of oxidative metabolism carried out to predict endpoints seen in PD development (Cloutier & Wellstead, 2012). We have also gained insights from a sophisticated stochastic modeling analysis of damaged mtDNA in aging mitochondria (Tam *et al*., 2015), where an OXPHOS defect threshold between 60% and 90% determined a deleterious senescence state with poor mitochondrial quality control. We built upon these original constructs and greatly expanded the focus to create a more complete view of the conserved cellular mechanisms implicated in biological aging. In doing so, we made a variety of assumptions and simplifications in our theoretical model to preserve the significance of the pathways involved and to arrive at a network that is mathematically logical. Importantly, the simulation assumes an isothermal environment (20 °C) for the duration of each virtual experiment, as *C. elegans* are poikilotherms and thus cannot properly adapt to drastic changes in temperature. Additional simplifications involve the condensed inclusion of conserved transcription factors DAF‐16 (FOXO family *C. elegans* ortholog) and SKN‐1 (orthologous to mammalian NRF family proteins), both of which have had profound implications in regulating longevity. When activated, DAF‐16 and SKN‐1 are largely responsible for mediating metabolic signals and antioxidant activities, and they have also been closely linked to the UPR^mt^ and mitophagy regulation (Park *et al*., 2009; Robida‐Stubbs *et al*., 2012; Mouchiroud *et al*., 2013; Palikaras *et al*., 2015). As the focus of this study is on mitochondrial function, these two transcription factors were explicitly embedded into the biological network to account mainly for the responses they elicit that significantly affect mitochondrial populations.

To make functional use of our cellular simulation, we have designed equations to capture key endpoints that have been observed quantitatively or qualitatively in aging *C. elegans*. Said endpoints include relative NAD^+^ levels, oxygen consumption rates, ATP content, tissue‐level and single‐cell mtROS content, tissue‐level and single‐cell mtDNA/∆mtDNA copy numbers, mitochondrial stress states and mitophagy flux, mitochondria count, and age‐dependent cellular senescence states and cell death (Box S6). The time frame simulated was between 0 and 30 000 min, to assess a window of time surrounding the average nematode lifespan of 2–3 weeks.

### Model performance and predictions for spontaneous functional declines

**Figure 2:**
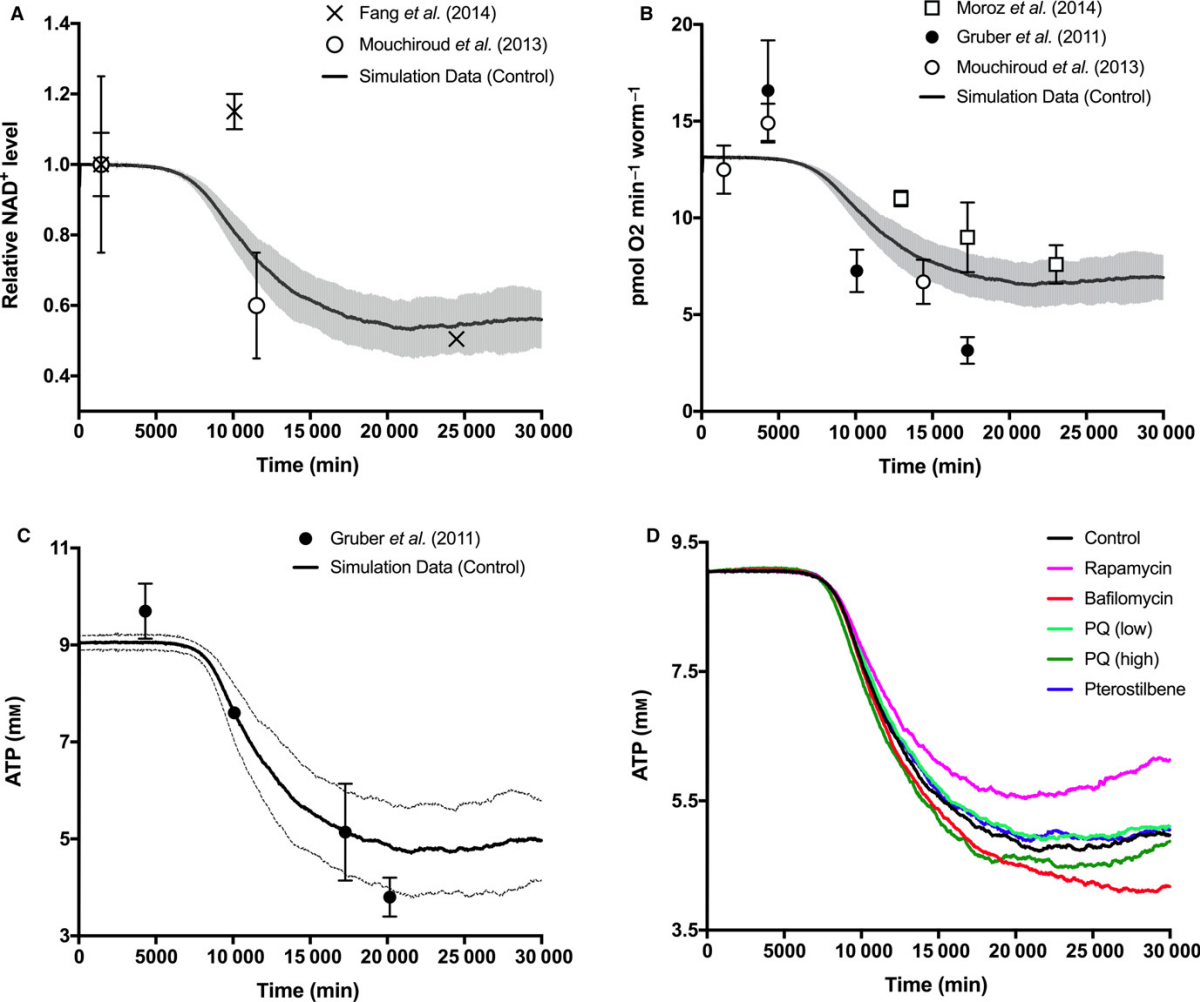
Experimental and predictive biomarkers of bioenergetic function for normal and pharmacologically altered aging in *Caenorhabditis elegans*. (A) Predicted NAD ^+^ values over time relative to initial time point values, compared to *C. elegans* experimental data from Fang *et al*. (2014) and Mouchiroud *et al*. (2013). (B) Simulated oxygen consumption decline with age, compared to *C. elegans* experimental data from Mouchiroud *et al*. (2013), Gruber *et al*. (2011), and Moroz *et al*. (2014). Some experimental oxygen consumption values were converted assuming an average protein mass per worm of 0.681 ± 0.002 μg. (C**)** Simulation ATP data for normal aging, compared to ATP levels shown experimentally in aging *C. elegans* by Gruber *et al*. (2011). Experimental ATP values were converted assuming an average worm body volume of 3.5 × 10^−9^ L. (D) Simulation ATP data were collected for the duration of natural aging as well as a variety of dosing schemes, including those that increased ATP production (rapamycin, pterostilbene, low‐dose paraquat) and those that decreased ATP production (bafilomycin, high‐dose paraquat).

Cellular characteristics of aging are all directly associated with a general decrease in cellular performance and are predominately regulated by OXPHOS functionality and overall mitochondrial health – especially in tissues with high energy demands. These functional declines ultimately manifest as decreases in ATP production, which have been ascribed to a variety of factors but the most significant of which include faulty matrix proteins and decreased metabolic substrate availabilities (Imai & Guarente, 2014). As a key substrate needed for respiration and other metabolic functions, NAD^+^ has been closely studied as an important molecule in healthy aging and has shown to decline in whole organisms over time (Mouchiroud *et al*., 2013; Fang *et al*., 2014). As such, nucleotide dynamics have been incorporated into our simulation constructs as a critical mediator of bioenergetic function, and we have with reasonable uncertainty predicted the relative declines that have been observed in *C. elegans* experimentally (Fig. 2A). As an additional measurable parameter of mitochondrial health and bioenergetic efficiency, oxygen consumption rates were calculated by the simulation over the course of a nematode lifespan and were compared to experimental datasets of the same decline generated by Gruber *et al*. (2011), Moroz *et al*. (2014), and Mouchiroud *et al*. (2013) (Fig. 2B). Some experimental oxygen consumption values were converted to units of pmol min^−1^ per worm assuming an average protein mass per worm of 0.681 ± 0.002 μg (Table S1). As an ultimate output of the bioenergetic simulation component, cellular ATP levels were assessed and compared to values quantified in whole worms by Gruber *et al*. (2011) (Fig. 2C). The concentration values generated by Gruber *et al*. (2011) have been converted to units of mm assuming an average worm body volume of 3.5 × 10^−9 ^L (Table S1). In the model, a sharp decline in tissue ATP presents itself after NAD^+^ levels have begun to decline (around 7500 min). ATP reduction then saturates around 20 000 min and becomes erratic toward the end of life due to the battery of activated stress responses in age‐compromised cells.

**Figure 3:**
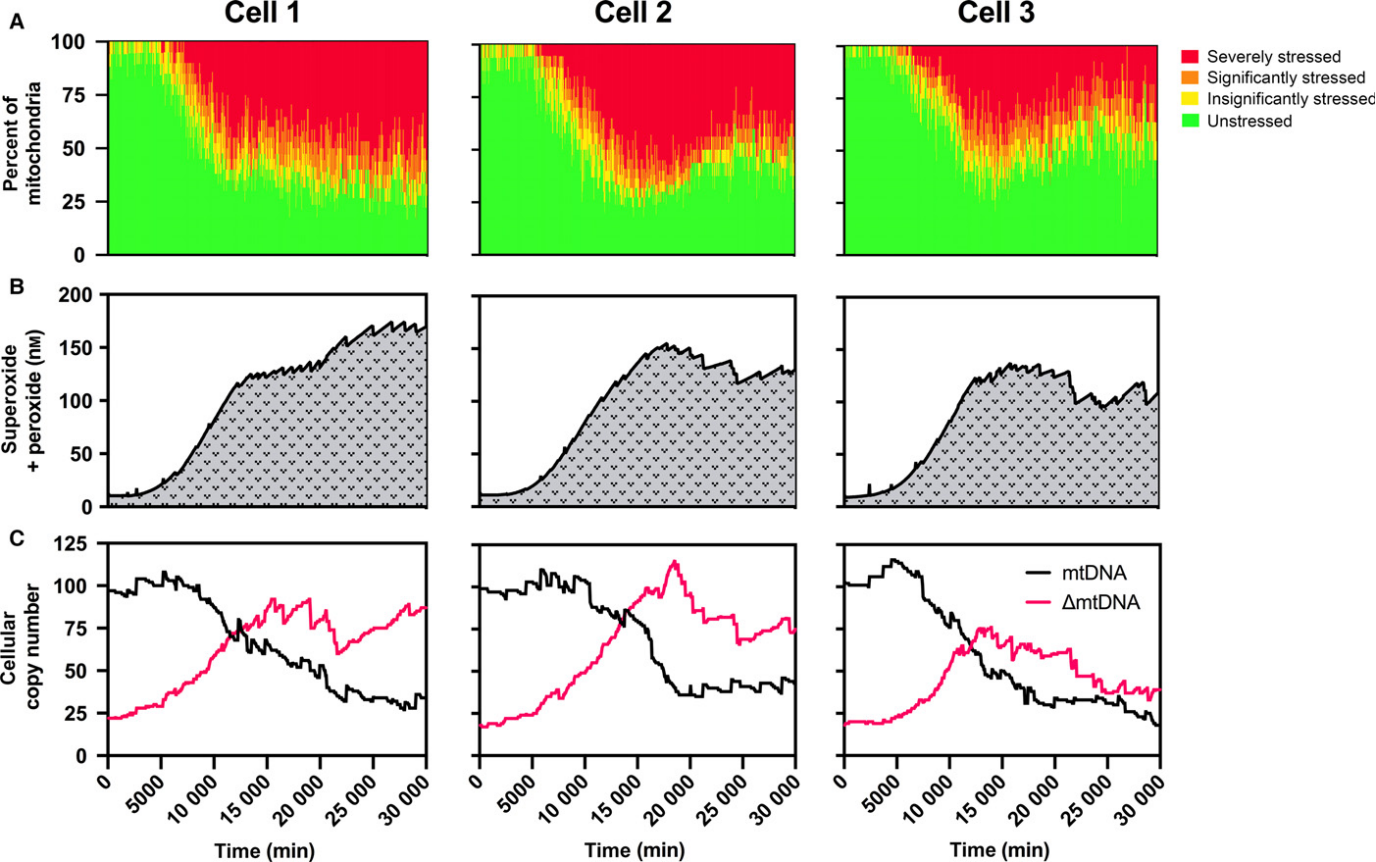
Stochastic single‐cell mitochondrial health data from three representative cells in the model. (Row A) Mitochondrial stress‐state phenotyping was performed to view the accumulation of stressed or damaged mitochondria over time. Different states of stress are based on metrics of mitochondrial dysfunction, including low respiratory capacity, oxidative burden and poor proteostasis. Some cells saw a steady decline in mitochondrial function (represented by cell 1), whereas others were able to successfully recover significant portions of their mitochondrial populations (represented by cells 2 and 3). (Row B) Single‐cell mitochondrial reactive oxygen species content was captured, which inversely correlated with the corresponding phenotypic trends in mitochondrial health. (Row C) Single‐cell mtDNA and damaged mtDNA (∆mtDNA) copies were analyzed, where high mutation and damage accumulation corresponded with highly dysfunctional phenotypes.

Because of the hierarchical modeling scheme employed here, we were able to analyze markers of aging both at the tissue level and in single cells. Specifically, we produced quantitative predictions of single‐cell mtROS and heteroplasmic mtDNA content. The model was also designed to phenotypically categorize each individual mitochondrion based on its intramitochondrial OXPHOS defects. Displayed here are three representative cells from the simulated population (Fig. 3), each displaying unique characteristics – due to the stochastic modeling techniques used to emulate a real‐world degree of expected randomness. Mitochondrial phenotyping (Fig. 3, row A), referred to hereafter as mito‐phenotyping, allowed us to visualize the totality of the cells’ mitochondrial stress states over time: healthy, fully functioning (green); slightly stressed, unable to greatly activate stress response mechanisms (yellow); significantly stressed, fully capable of stress response activation (orange); and severely damaged, little to no proper respiratory function (red). Some cells saw a steady functional decline with respect to this mito‐phenotyping (Fig. 3, cell 1), whereas others saw modest recovery toward late stages in life (Fig. 3, cells 2 and 3). These stress states correspond well with the levels of mtROS seen in each modeled cell (Fig. 3, row B). Of these representative cells, the third (cell 3) experienced the highest level of oxidative stressors early on, but also experienced the highest level of recovery. This corresponds with the hypothesis that superoxide or other free radical species may not be inherently damaging and may actually provide a signal that promotes longevity (Yang & Hekimi, 2010). Single‐cell mtDNA and significantly damaged mtDNA (∆mtDNA) copy numbers were also assessed in the simulation (Fig. 3, row C), displaying the age‐dependent accumulation of heteroplasmic mtDNA. As expected, the cells that accumulated higher copy numbers of ∆mtDNA faster – especially when far exceeding unperturbed mtDNA copy numbers – experienced the greatest deal of mitochondrial stress.

**Figure 4:**
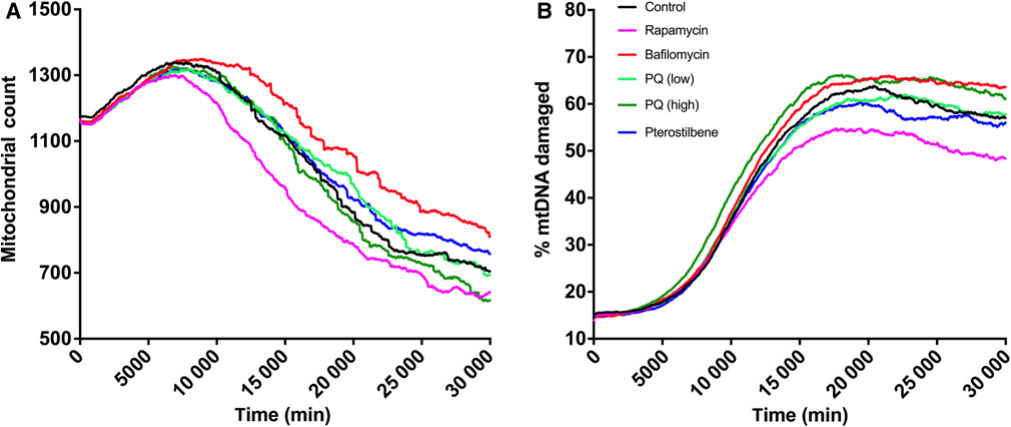
Model‐generated mitochondrial counts and heteroplasmic mtDNA content in normal and pharmacologically altered aging. (A) Mitochondrial counts were assessed in our control simulation and also in the same treatment groups as Fig. 2(B), displaying results that give rise to changes in aging mitochondrial dynamics, that is, mitophagy and mitochondrial recycling. (B) Percent of mtDNA damage was calculated in each simulation experiment as an important metric of age‐related mitochondrial dysfunction. Different perturbation schemes modulated the accumulation of mtDNA modifications, which when reduced has been implicated as a relevant intervention in combatting age‐related disease.

**Figure 5:**
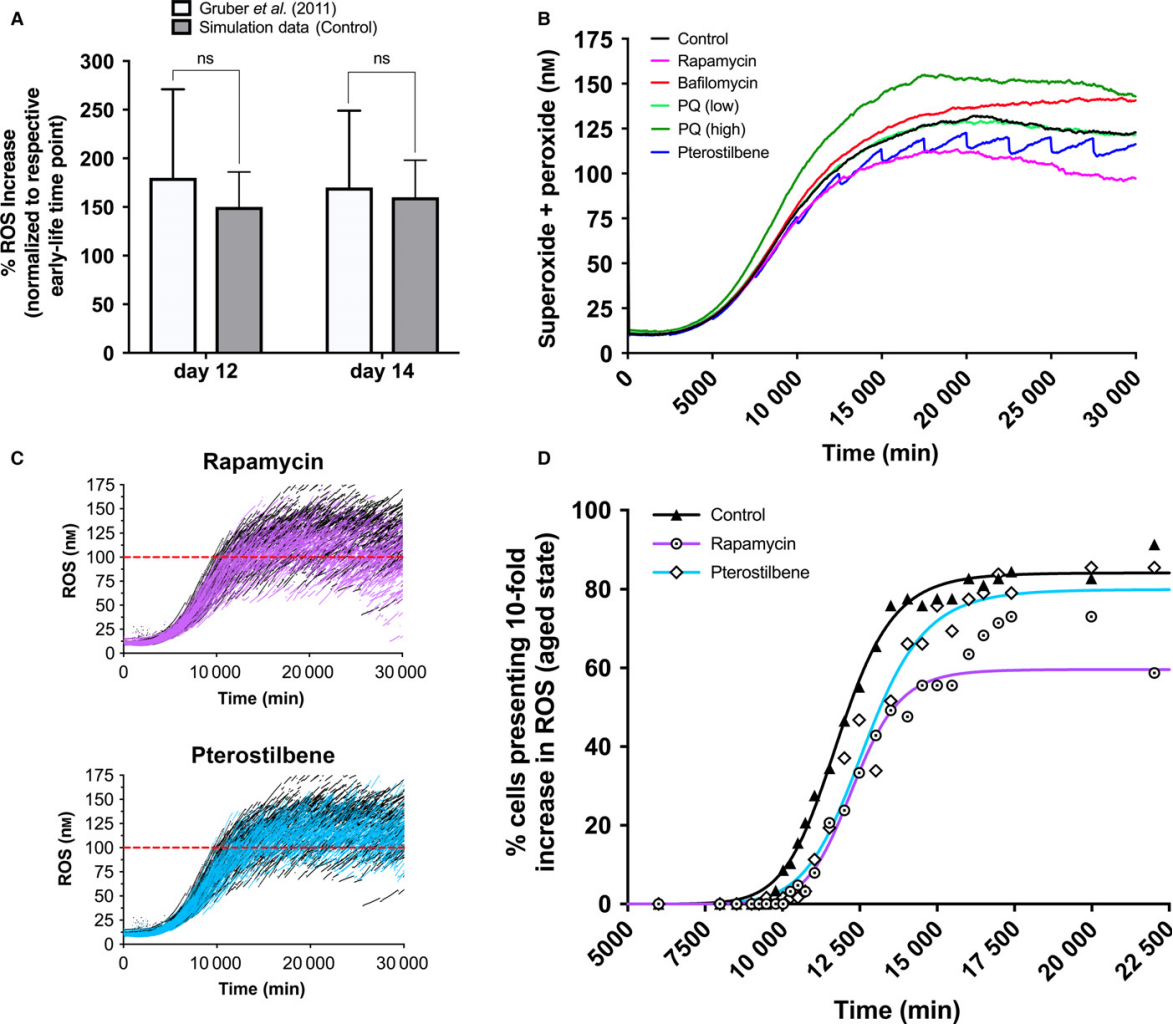
Simulation analysis of normal and pharmacologically modified oxidative burden in aging cells. (A) The simulation was able to reproduce age‐related increases in oxidative species as compared to *Caenorhabditis elegans* data from Gruber and colleagues (Gruber *et al*., 2011) (^ns^ *P* > 0.05). Increases within simulation or experimental data were normalized to their respective levels seen at the time point immediately preceding a fully aged state determined by the model (~day 7). (B) Average oxidative stressor levels, superoxide and peroxide, were determined in a variety of experiments (same treatments as Fig. 2B) for the population of cells simulated over the timeframe of interest. (C) Single‐cell reactive oxygen species (ROS) data were model‐generated for each individual cell in natural aging, in rapamycin‐exposed cells (top panel, overlaid with control), and in pterostilbene‐exposed cells (bottom panel, overlaid with control). These data signify probabilistic, rather than deterministic, mitochondrial activities within each cell giving rise to the natural stochasticity that determines composite ROS values for aging tissues. (D) Simulation time required for cells to switch to the age‐compromised state, characterized by a 10‐fold increase in ROS (100 nm, the approximate level of oxidative stress required to elicit an aged phenotype as determined by the model).

Mitochondrial dynamics, including biogenesis and selective mitophagy, were simulated, and organelle counts were tracked for an entire population of cells (Fig. 4A). Natural aging (control) allowed for a modest increase in mitochondrial content until a midlife stage, and counts significantly declined until the end of life. This is consistent with previously published trends seen in *C. elegans* (Palikaras *et al*., 2015), concerning both general cellular activity as well as neuron‐specific mitochondrial tracking. In the simulated cell population, mtDNA and ∆mtDNA content were tracked and presented as a deleterious heteroplasmic mtDNA percentage (Fig. 4B). As with other makers of age‐related damage, mtDNA copies experienced steady and significant damage with normal aging (control), and the damage percentage saw a slight, yet erratic, decline in late stages of life due to natural defense mechanisms. Oxidative stress levels were measured *in silico* as well (Fig. 5A), and closely matched spontaneous increases seen previously in *C. elegans* (Gruber *et al*., 2011). Experimental and simulation values were both normalized to the respective oxidative stress levels immediately preceding the simulated age‐compromised state (day 7).

While the network motifs employed in our simulation allow us to reproduce the levels of certain age‐dependent respiratory function markers, a sensitivity analysis was also conducted to determine which model functions and constants provide the strongest control over the system. The results of this analysis are tabulated in Table S4 (Supporting Information), where ROS outputs and macromolecule damage levels were sensitively controlled by many different parameters at various ages. Perhaps most notably, mitophagy activity and mitochondrial content at a late age time point were deemed to be the most sensitively controlled by many parameters of the model.

### Endpoint changes after control of selective mitophagy

To perturb the computational model, we determined mathematical methods for introducing rapamycin or bafilomycin as regulators of mitophagy (Box S7). Such methods included the estimated dose–response curve for autophagy activation/inhibition as well as the culture pharmacokinetics for determining the presence of each agent. Rapamycin is a well‐known inhibitor of the mechanistic target of rapamycin (mTOR) complex (Robida‐Stubbs *et al*., 2012), which ultimately allows for increased autophagic activity (Fig. 1; see Rapa). Bafilomycin, on the other hand, inhibits the formation of autophagosomes and therefore inhibits general autophagy and selective mitophagy (Mauro‐Lizcano *et al*., 2015) (Fig. 1; see Baf). In our simulation, constant low‐level dosing of each of these compounds (15 nm rapamycin or 10 nm bafilomycin, refreshed every 2500 min) was used in these comparative perturbations. As expected, rapamycin (pink line) curbed the decline of ATP production in aging cells within the simulation experiments, whereas bafilomycin (red line) exacerbated this specific decline in energy production (Fig. 2D). Demonstrating that our mathematical perturbations were successful in emulating the function of pharmacological mitophagy modulators, rapamycin and bafilomycin elicited drastic decreases and increases in mitochondrial count compared to the control, respectively (Fig. 4A). In addition, accumulation of deleterious heteroplasmic mtDNA was initiated sooner by bafilomycin exposure and was stunted by exposure to rapamycin (Fig. 4B). Rapamycin was used previously to investigate this exact effect, where it was shown to attenuate the accumulation of mtDNA harboring a specific pathogenic mutation (Dai *et al*., 2014). As a result of harboring defective mitochondria, simulated mtROS levels were perturbed by these mitophagy modulators as well, where rapamycin‐exposed cells saw less oxidative stress with age and bafilomycin‐exposed cells saw much more (Fig. 5B).

### Direct and indirect modulation of age‐dependent oxidative stress levels

To target alternative pathways involved in our *in silico* cellular mechanism, we chose two particular agents known to modulate mitochondrial processes associated with age‐dependent oxidative damage: paraquat and pterostilbene (Box S7). Paraquat is a well‐established potent ETC uncoupling agent (Park *et al*., 2009) that causes profound increases in oxidative stress (Fig. 1; see PQ). Pterostilbene is best known as a constituent of certain berries and red wine and as a relative of resveratrol, a more commonly known stilbenoid compound. In contrast to paraquat, exposure to pterostilbene or synthetic relatives has shown to indirectly decrease oxidative stress in *C. elegans*, in part by contributing to the activation of DAF‐16 and SKN‐1 (Fischer *et al*., 2017) (Fig. 1; see PT). In our simulation, constant dosing of 100 μm pterostilbene and 5 or 100 μm paraquat was used in these comparative perturbations. Interestingly, ATP content was considerably reduced by high‐dose paraquat but slightly improved by low‐dose paraquat and pterostilbene treatments (Fig. 2D). Compared to the control, low‐dose (light green line) and high‐dose (dark green line) paraquat caused increases and decreases in mitochondrial counts, respectively (Fig. 4A), and only high‐dose paraquat enabled a greater accumulation of ∆mtDNA (Fig. 4B). A similar mtROS‐dependent effect has been shown experimentally for mtDNA double strand breaks causing accelerated aging in mice (Pinto *et al*., 2017). Virtual pterostilbene exposure (blue line) elicited marginally less mitochondrial turnover toward late age (Fig. 4A). This finding is surprising as stilbene compounds have been suggested as agents that promote autophagy (Poulose, 2015), but perhaps not with selective and nonselective mitophagy. Pterostilbene was the only compound that prolonged the accumulation of ∆mtDNA even though it eventually reached the same percentage as the control (Fig. 4B). This highlights the interesting aging response phenomena associated with stilbene compounds, especially with those that potently affect intramitochondrial reaction networks.

Indicating our mathematics were successful in emulating the toxicological uncoupling effect at the ETC, low‐dose paraquat resulted in very slight increases in oxidative stress, whereas the higher dosing scheme prompted a drastic surge in both superoxide and peroxide production (Fig. 5B). It is important to note that although low‐dose paraquat initially causes increases in mtROS, it also at times caused reductions compared to control at late time points. A similar reduction in mtROS was also observed in our simulation after ablating *sod‐2* (MnSOD) at increasing levels (Fig. S3A,B). Consistent with previously published nematode data (Fischer *et al*., 2017), pterostilbene administration in our analysis allowed for around a 20–25% reduction (slowly reversible) in oxidative stress at the maximal response time points in the model after reaching fully aged states (Fig. 5B). As pterostilbene and rapamycin showed the most promising results for slowing the damaging effects of aging mitochondria, we analyzed their oxidative stress modulation at the single‐cell level. Our computational model allowed us to visualize mtROS levels over time overlaid with control data for a population of cells (*n* = 65; 500 time points), demonstrating both the highly stochastic nature of the model and the protective effects of rapamycin (Fig. 5C, top panel) and pterostilbene (Fig. 5C, bottom panel). To distill this information, we determined that 100 nm combined superoxide and peroxide allowed for a deleteriously aged cellular phenotype and assessed how many cells crossed this threshold at various time points for natural aging (control), rapamycin, and pterostilbene (Fig. 5D). Pterostilbene and rapamycin greatly extended the time required for the majority of cells to cross this threshold; however, rapamycin further reduced the number of total cells that experienced this aged phenotype.

### Cell death predictions and implications for age‐related neurodegeneration

**Figure 6:**
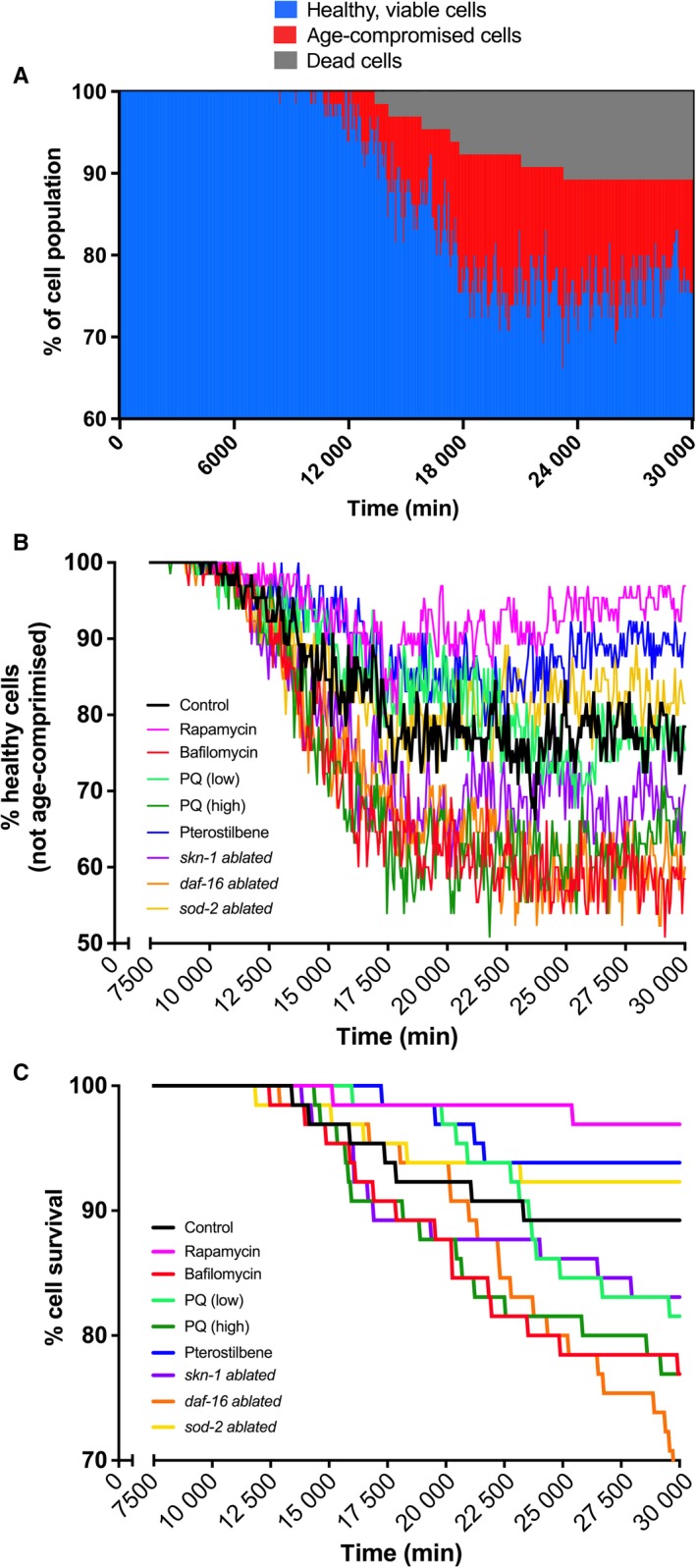
Age‐dependent total cellular damage and degeneration analysis. (A) Natural aging, presumably as a result of faulty mitochondrial dynamics, forced healthy cells (blue) into a compromised state (red) in our simulation, where they were required to overcome mitochondrial defects in order to recover. If recovery was insufficient, they died off (gray). (B) The dynamics of natural age‐compromised cell accumulation (control) was compared to those virtually altered by pharmacological (rapamycin, bafilomycin, paraquat, or pterostilbene exposure) and genetic (*daf‐16*,* skn‐1*, or *sod‐1* ablation) simulation schemes. (C) Natural cell survival rates were compared to the same perturbation schemes of interest. Many schemes caused cells to age faster in the simulation, resulting in more death early on. Some simulation perturbations, however, were able to slow the rate of aging and natural degeneration, either slightly or robustly.

Given that aging and senescence are implicated in various cognitive deficits and neurodegenerative diseases, we used our computational methods to ultimately simulate an age‐compromised cell state and subsequent probabilistic death for postmitotic energetically demanding cells (Fig. 6A). The rate of spontaneous degeneration simulated here corresponds well with wide speculations made about the slow loss of neuronal mass that accompanies old age in mammals (Peters, 2006). This degenerative response has been more complex to quantitate in nematodes, as the neuronal network appears to be conserved with age (Herndon *et al*., 2002) yet vacuolization and cellular damage certainly occur (Hall *et al*., 1997). Cells within the model began to enter the compromised state around 10 000 min, shortly after cells began crossing the model‐determined mtROS threshold (10‐fold increase) as mentioned in the previous section and shown in Fig. 5D. Viable cell and cell survival curves, associated with age‐compromised cell states and degenerative aging, were generated for a battery of *in silico* mechanistic perturbations, including the pharmacological treatments of interest as well as reductions in *sod‐2*,* daf‐16* and *skn‐1* expressions (Fig. 6B,C). Pterostilbene exposure (blue lines) rapamycin exposure (pink lines), low‐dose paraquat exposure (light greens) and 90% *sod‐2* ablation (yellow lines) all delayed immersion into compromised states and extended cellular longevity, with rapamycin and pterostilbene eliciting the strongest effects. The remaining treatment groups, bafilomycin (red lines) high‐dose paraquat (dark green lines), 90% *daf‐16* ablation (orange lines) and *skn‐1* ablation (purple lines), all resulted in rapid aging and severely decreased survival rates. While *sod‐2* ablation resulted in a marginally beneficial response, our simulation results indicate that its expression must be fully reduced to see any increases in longevity‐promoting effects (Fig. S3C,D), due to the stress responses that the increased endogenous mtROS activate. This aging delay brought on by the mathematical model substantiates previous studies that have linked increases in oxidative species to extended lifespans (Yang & Hekimi, 2010; Gruber *et al*., 2011); however, the timing and duration of genetically or pharmacologically induced mtROS must play a critical role in regulating these mechanisms as well.

## Discussion

Biological aging is the primary phenomenon governing organismal fitness and has often been deemed the most important risk factor in debilitating and fatal diseases. Age‐dependent diseases that are degenerative or senescence‐controlled in nature are generally confined to tissues with high energy demands, such as skeletal and cardiac muscle and nervous tissue (Niccoli & Partridge, 2012). These incidences suggest that severe mitochondrial dysfunction and aging are not mutually exclusive processes. The fundamental causalities between the two, however, are considerably complex and challenging to investigate, denoting the ongoing need for innovative and powerful methods in aging research. To provide an additional and systematic means of investigation, we have designed a computational network that integrates many of the sophisticated experiments completed in recent years. By balancing spontaneous free radical damage as well as corresponding mitochondrial stress responses, we have been able to make apt predictions about the aging process in a well‐studied model organism. Several of our large‐dataset predictions have been validated quantitatively (Figs 2A–C and 5A) or qualitatively, while several have yet to be experimentally examined, leaving well‐designed hypotheses for future work.

As part of our modeling study, we sought to understand how crucial the UPR^mt^ and mitophagy are in balancing the oxidative stress and macromolecule damage that accompany normal, healthy aging. It was revealed in our experiments and global sensitivity analysis that mitochondrial turnover and quality control levels were the most important processes in either softening or exacerbating the deleterious effects of mitochondrial dysfunction in aging. This is substantiated by previous experiments carried out in *C. elegans*, which suggest the UPR^mt^ is not a potent regulator of longevity pathways (Bennett *et al*., 2014), whereas mitophagy likely is (Palikaras & Tavernarakis, 2012). The UPR^mt^, although not apparently important in regulating the lifespan of a full organism, may play a critical role in the cellular aging mechanisms that regulate tissue‐specific healthspans. This is especially true for sensitive tissues with high mitochondrial counts, as they have a direr need to quickly regulate their antioxidant defenses and mitochondrial protein environments. Adding to this complexity, activation of this response must be performed carefully, and likely on a low‐frequency basis, as the UPR^mt^ has been implicated as a mechanism that can protect aberrant mitochondrial contents (Lin *et al*., 2016). In particular, overactivation of this response has been linked to a higher persistence of mtDNA heteroplasmy. The mechanisms by which this occurs are poorly understood, although they are not entirely dependent upon mitophagy. However, mitophagy may certainly play a small role in the UPR^mt^'s ability to maintain defective mitochondria, as TIMM‐23 expression is upregulated by the UPR^mt^ and is responsible for the import and degradation of pro‐mitophagy proteins such as PINK‐1 (Menzies *et al*., 2015). Therefore, an overexpression of TIMM‐23 may limit, for a short duration of time, the amount of mitophagy activity. In contrast to this theory, it has been shown that the complete absence of TIMM‐23 isoforms constitutively induces the UPR^mt^ and leads to decreases in lifespan (Bennett *et al*., 2014). These observations exemplify the physiological need for an incredibly tight regulation of TIMM‐23‐dependent transport processes and mitophagic activities that may be programmed to vary with age in an attempt to maximize mitochondrial healthspans.

In addition to gaining mechanistic insights, we used our multimethod simulation to predict the cellular effects brought on by different pharmacological agents that have been widely studied as modulators of aging, especially in neuronal tissue. Most agents that have been discovered as longevity‐promoters have also been known to promote mitochondrial health. Rapamycin exposure has led to stark increases in stress resistance to age‐dependent damage in a wide variety of organisms (Robida‐Stubbs *et al*., 2012; Kolosova *et al*., 2013; Martínez‐Cisuelo *et al*., 2016), presumably due to its regulatory role in metabolic processes and nonselective autophagy. In our simulation experiments, we applied it as a mitophagy inducer and observed remarkable decreases in heteroplasmic mtDNA content and increases in antioxidant capacity. Both of these rapamycin‐induced effects have been observed experimentally (Robida‐Stubbs *et al*., 2012; Dai *et al*., 2014). Previous experiments and our simulation work draw attention to rapamycin as not only an agent that can extend the lifespan of an organism, but as a compound that does so by preserving the quality of mitochondria. Plant‐derived polyphenols, such as resveratrol and pterostilbene, have also been studied for their potential ability to stunt the aging process through antioxidant activities and mitochondrial pathways. In *C. elegans*, resveratrol has been shown to alleviate oxidative stress and resulting mitochondrial damage associated with respiratory chain deficiencies, resulting in extended lifespan (McCormack *et al*., 2015). Although a significant portion of the literature has documented the longevity‐promoting effects of resveratrol, much of the recent focus has fallen on pterostilbene and synthetic derivatives, as they have been observed as more potent compounds capable of modulating cellular processes in aging and age‐related neurodegeneration (Chang *et al*., 2012; Fischer *et al*., 2017), in part through the DAF‐16 and SKN‐1 axes. For this reason, we chose pterostilbene as the virtual stilbene compound to be used in our simulation work. Based on dose–response data that allowed us to calculate its mtROS remediation (Fischer *et al*., 2017), pterostilbene prolonged mitochondrial damage accumulation and age‐dependent cellular degeneration in our simulations, albeit to a lesser extent than rapamycin. Notably, pterostilbene acts by increasing the activity of NAD^+^‐dependent deacetylases known as sirtuins (Imai & Guarente, 2014), which promote well‐balanced oxidative metabolism within mitochondria, and particular isoforms are involved in activating the UPR^mt^ (Mouchiroud *et al*., 2013; Papa & Germain, 2014). If the UPR^mt^ and mild antioxidant defenses are not as powerful anti‐aging contributors as mitophagy and mitochondrial biogenesis, this explains the more favorable simulation results returned for rapamycin. Pterostilbene, however, has been known to induce autophagy on some level (Poulose, 2015), and NAD^+^‐dependent sirtuin activities are important in regulating not only bioenergetic functions and antioxidant capacity but mitochondrial dynamics as well (Fang *et al*., 2014); therefore, it must be investigated whether this compound can effectively stimulate the process of selective mitophagy in relevant cell types to promote significant increases in mitochondrial quality.

Computational modeling has recently become a more commonplace technique in gerontological research, with many different types of developed models surfacing over the past decade (Mooney *et al*., 2016). Such models include purely deterministic analyses of oxidative metabolism (Cloutier & Wellstead, 2012), well‐developed Boolean approaches for understanding molecular aging networks (Verlingue *et al*., 2016), discrete‐event modeling of mtDNA dynamics (Tam *et al*., 2015), and high‐precision statistical modeling of lifespan determinants (Stroustrup *et al*., 2016).

Here we have contributed a new multimethod computational modeling technique for studying the cellular mechanisms of aging as they relate to mitochondrial function and dysfunction. We have built the groundwork for a comprehensive computational model that can reproduce levels of mitochondrial and cellular biomarkers implicated in aging, with respect to *C. elegans*. In particular, our model has highlighted the potent effects of (i) mitophagy induction or inhibition and (ii) the critical stress response genes, such as *daf‐16* and *skn‐1*, required to maintain healthy aging and to further promote longevity through oxidative mechanisms. After further mechanism integration and experimental interrogation, this computationally intensive tool may be able to accurately predict aging phenotypes and neurodegenerative disease markers – first in nematodes and eventually in larger, more complex organisms. In addition, future *in silico* interrogations will provide a powerful, quick, high‐throughput, and cost‐effective means by which to test experimental drugs and dietary supplements that may very well slow the deleterious effects of aging and the pathogenesis of age‐related diseases.

## Experimental procedures

### Hierarchical cell simulation design and agent‐based modeling

To simulate intramitochondrial system dynamics and global cellular reactions, we formulated our model using AnyLogic^®^ multimethod simulation software (The AnyLogic Company, Chicago, IL, USA), which allowed us to develop a hierarchical agent‐based simulation scheme. This allowed us to simulate a population of cell agents, with each individual cell agent containing its own population of mitochondrion agents (Fig. 1B). Each agent contained discrete‐event statements and system‐dynamics reaction networks, both of which were programmed to deal with natural biological stochasticity. As a critical agent‐based element employed in our model, cells harboring a high percentage of faulty mitochondria were forced into a compromised state via a discrete event. These types of events can be activated once a particular condition is met, and in this case, the event was triggered once enough mitochondria were severely damaged and defective. As this percentage trigger is presumably different for each cell, we incorporated a stochastic transition statement that allowed each cell to pull from a triangular probability:

$$\begin{array}{cc} & \text{Condition}:(\mathrm{Defective}\_\mathrm{Mitochondria}/\mathrm{TotalMitoCount})\\ & >\mathrm{triangular}\;(0.60,0.625,0.75). \end{array}$$

The range is denoted by the outer bounds and the most probable value is denoted by the middle value of 65%. Similar agent‐based principles were utilized for mitochondrial stress‐state transitions and are fully outlined in the Supporting Information.

### Deterministic modeling components

Many biological events have been proposed as crucial elements in regulating cellular and organismal lifespan. We manually selected and organized several such elements from the literature and implemented them in complement to our agent‐based work as a deterministic modeling framework, that is, an intricate web of molecule‐dependent ODEs. Such elements included (i) mitochondrial reaction networks that govern oxidative metabolism, proteostasis, and genomic alterations and (ii) global cellular reaction networks pertaining to ATFS‐1 movement in the cell, protein synthesis, and mitochondrial import. The majority of these curated reactions are detailed in Fig. 1(A), and all of the equations and corresponding parameters used are organized and tabulated in the Supporting Information.

### Sensitivity analysis

To determine the quantitative significance of certain parameters and functions within the system, a similar global sensitivity strategy was used that has been described previously (Tam *et al*., 2015). Briefly, each parameter was individually reduced by 5%, and the resulting model outputs, referred to in the text as response variables, were assessed. This was done for all static parameters within the model, and the changes in several response variables (e.g., ROS output) were measured at particular time points determined for young age, midlife, and old age. Sensitivity coefficients for each scenario were calculated:

$$\text{SC}=\left(\frac{\partial O_{i}}{\partial P_{j}}\right)\times \left(\frac{P_{j}}{O_{i}}\right)=\left(\frac{\partial O_{i}}{O_{i}}\right)\times \;20,$$

where SC represents the normalized sensitivity coefficient, *O*_*i*_ is the model output value, *P*_*j*_ is the value of the parameter of interest, and ∂ represents the partial derivative of either the parameter value or model output. When |SC| ≥ 1.0000, that is, when the proportional change in the response variable is greater than the proportional change in the parameter, the parameter is deemed to have significant sensitive control over the respective output at the specified age.

### Statistical analysis

Statistical significance was determined by using GraphPad Prism 7.0 software, and *P* values were calculated by a one‐way analysis of variance (ANOVA). Significance was considered to be a *P* value of 0.05.

## Author contributions

WHH contributed the software packages and analytical tools. TEH and WHH conceived the theories and hypotheses prior to *in silico* experimentation. TEH, WHH, and LW developed the methodologies and simulation experiments. TEH performed the research experiments and analyzed the data. WHH, LW, and KJB examined the data and provided significant feedback. TEH, WHH, KJB, and LW contributed significantly to the preparation of this manuscript.

## Funding

This work was supported by the Center for Environmental Medicine at Colorado State University.

## Conflict of Interest

The authors declare no conflict of interest.

## Supporting information


**Fig. S1** Schematics of mitochondrion‐level modeling dynamics.
**Fig. S2** Schematics of cell‐level modeling dynamics.
**Fig. S3** MnSOD simulation experiments for varying gene expression levels
**Table S1** Cellular and anatomical parameters of interest for *C. elegans*.
**Table S2** Parameters of interest for the proposed intramitochondrial system.
**Table S3** Parameters of interest for the proposed intracellular system.
**Table S4** Normalized sensitivity coefficients against respective response variables.
**Box S1** Equations pertaining to the estimation of nematode‐specific cellular parameters.
**Box S2** Equations and event statements pertaining to mitochondrion‐level kinetics.
**Box S3** Conditional and rate‐dependent statements governing the rule‐based categorization of mitochondrial stress, via the agent‐based paradigm.
**Box S4** Equations pertaining to cell‐level kinetics.
**Box S5** Conditional and rate‐dependent statements governing the rule‐based categorization of cell integrity, via the agent‐based paradigm.
**Box S6** Equations used to capture tissue concentrations of endpoints of interest.
**Box S7** Functions used to define virtual pharmacological perturbations within the model.
