# Supplementary material for: A multimethod computational simulation approach for investigating mitochondrial dynamics and dysfunction in degenerative aging
Source: Aging Cell. 2017 Aug 16;16(6):1244–55. doi: 10.1111/acel.12644 (PMC5676065; doi:10.1111/acel.12644)
Supplement: Supplementary file 1 — Fig. S1 Schematics of mitochondrion‐level modeling dynamics. Fig. S2 Schematics of cell‐level modeling dynamics. Fig. S3 MnSOD simulation experiments for varying gene expression levels Table S1 Cellular and anatomical parameters of interest for C. elegans. Table S2 Parameters of interest for the proposed intramitochondrial system. Table S3 Parameters of interest for the proposed intracellular system. Table S4 Normalized sensitivity coefficients against respective response variables. Box S1 Equations pertaining to the estimation of nematode‐specific cellular parameters. Box S2 Equations and event statements pertaining to mitochondrion‐level kinetics. Box S3 Conditional and rate‐dependent statements governing the rule‐based categorization of mitochondrial stress, via the agent‐based paradigm. Box S4 Equations pertaining to cell‐level kinetics. Box S5 Conditional and rate‐dependent statements governing the rule‐based categorization of cell integrity, via the agent‐based paradigm. Box S6 Equations used to capture tissue concentrations of endpoints of interest. Box S7 Functions used to define virtual pharmacological perturbations within the model. [file ACEL-16-1244-s001.pdf]

Supporting Information

A Multimethod Computational Simulation Approach for Investigating Mitochondrial Dynamics and Dysfunction in Degenerative Aging

TEH, KJB, LW, WHH (2017)

COMPUTATIONAL SIMULATION METHODS
Tables S1 – S4
Boxes S1 – S7
Figures S1 – S3

General model units and organism-specific cellular organization parameters

The model is designed to survey a population of 65 neuron-like cells, each with its own population of functioning mitochondria. This cell number was chosen to produce a large enough population to observe without exceeding the limitations of the simulation software, as the methodology is computationally very intensive. The mitochondrial count within each cell varies between 5–35 organelles as determined by literature-based calculations (Table S1; Box S1). Since the cells will attempt to emulate the energy demand of neurons or myocytes within *C. elegans*, we chose a mean mitochondrial count of 15 with a Poisson distribution to introduce truer cellular stochasticity.

Table S1. Cellular and anatomical parameters of interest for 3-day-old C. elegans (N2); ranged/averaged literature values.

Parameter	Symbol	Value Range	Units	Source
Average body volume	*Bv*	3.5 10^{-9}	L/worm	[1]
Average body density (young adult)	*Bd*	1081 ± 3	mg/L	[59]
mtDNA per mitochondria	*MmtDNA*	2 – 10 (rat)	mtDNA-count/mito	[2]
Average mtDNA per worm	*WmtDNA*	250,000 – 320,000	mtDNA-count/worm	[3]
Estimated mtDNA copies per cell	*CmtDNA*	45 – 70	mtDNA-count/cell	[4]
Mitochondria range per worm	*wMito*	25,000 – 160,000	mito-count/worm	calculated
Mitochondrial range per cell	*cMito*	5 – 35	mito-count/cell	calculated
Average ATP per worm	*wATP*	30 – 40	pmol/worm	[3]
Cellular ATP concentration	*cATP*	2.86 – 8.57	mM	calculated

Box S1: Equations pertaining to the estimation of nematode-specific cellular parameters.

Mitochondria range per worm: $$wMito = \frac{WmtDNA}{MmtDNA}$$

Mitochondria range per cell: $$cMito = \frac{CmtDNA}{MmtDNA}$$

ATP concentration range: $$cATP = \frac{wATP}{Bv}$$

Average protein mass per worm: $$WormPMass = Bd * Bv * 18\% \left(\frac{protein}{total\ mass}\right) = 0.681 \pm 0.002\ \frac{\mu g_{protein}}{worm}$$

Table S2. Parameters of interest for the proposed intramitochondrial biochemical system.

Parameter	Symbol	Value / Range	Units	Source
second-order rate constant for MnSOD-mediated superoxide transformation	*k1*	1.0×10^{-2}	$nM^{-1}\ min^{-1}$	[3][A,C]
average MnSOD conc. in mitochondria (WT)	*[MnSOD]$_o$*	$3.0 – 6.0 \times 10^{3}$	nM	[3,5][C]
aggregate first-order rate constant for other reactions yielding peroxide	*k2*	5.0	min^{-1}	[3][A,C]
aggregate first-order rate constant for reactions not yielding peroxide	*k3*	30.0	min^{-1}	[3][A,C]
zero-order superoxide generation rate	*v4*	$4.0 – 24.0 \times 10^{4}$	$nM\ min^{-1}$	[3,6][A,C]
constant for feedback inhibition by superoxide	*Ki*	7.8×10^{-2}	nM	[3][C]
first-order rate constant for peroxide disposition	*k5*	0.38	min^{-1}	[7][SS]
initial total concentration of functional ETC proteins	*$oEP_{functional}$*	100	%	fixed assumption
initial total concentration of faulty ETC proteins	*oEP_{faulty}*	1	%	fixed assumption
initial total concentration of unused ETC subunits	*oEP_{usable}*	100	%	fixed assumption
pseudo zero-order ADP production rate	*v6*	15.8 – 21.0	$mM\ min^{-1}$	[8][SS,C]
pseudo first-order complete elimination rate constant for ATP	*k7*	1.87	min^{-1}	[8][C]
pseudo zero-order rate of total mitochondrial ETC subunit protein synthesis	*v8*	$9.0 – 36. \times 10^{-4}$	$\%\ min^{-1}\ copy^{-1}$	[9][C]
second-order rate constant (with Kc) governing the protection/recovery of total mt-ETC proteins	*k9*	k10 (1/15)	min^{-1}	[10,11,12][A]
second-order rate constant (with Kros) for oxidation of usable ETC proteins	*k10*	0.1	min^{-1}	[13][A]
ROS-neutralizing component of the corresponding second-order rate constant for oxidative activity of [$O_2^{\cdot-}$] and [H_2O_2]	*Kros*	0.094	nM^{-1}	[3, 7][O]
second-order rate constant (with Kc) for the elimination of usable proteins via ETC incorporation	*k11*	k14 (¼)	min^{-1}	[14][A]
second-order rate constant (with Kp) for the elimination of functional ETC complexes via protein turnover	*k12*	$3.5 – 10.5 \times 10^{-5}$	min^{-1}	[15][C]
protein-neutralizing component of the corresponding second-order rate constant for chaperone activity	*Kc*	0.001	$\%^{-1}$	[3][O]
protein-neutralizing component of the corresponding second-order rate constant for protease activity	*Kp*	0.094	$\%^{-1}$	[3][O]
pseudo zero-order rate of total faulty mitochondrial protein synthesis from damaged mtDNA	*v13*	$9.0 – 36.0 \times 10^{-4}$	$\%\ min^{-1}\ copy^{-1}$	[9][C]
second-order degradation constant (with specific protease activity, Kp) for the elimination of deleteriously oxidized proteins	*k14*	1.7×10^{-2}	min^{-1}	[6][C]
rate variable governing oxidative damage to mtDNA	*damageRate*	0.070	$events\ mM\ week^{-1}$	[3, 16][O]
rate variable governing removal of ΔmtDNA	*destroyRate*	5.148	$events\ month^{-1}$	[3, 17][O]

[SS] Optimized in the simulation to achieve the reported unstressed physiological steady-state value, percentage or range.
[O] Individually optimized value, generated using AnyLogic calibration experiments to best fit or emulate reported kinetic curves.
[A] Quantitated assumption based on qualitative evidence.
[C] Units converted to adhere to model standards.

Box S2: Equations and event statements pertaining to mitochondrion-level biochemical kinetics.

Differential equations governing the time-dependent amounts of key players (PQ_{ROS} represents a function of paraquat-induced increases in ROS production rate, and PT represents a function of pterostilbene-mediated ROS mitigation, as outlined in the last section; when unperturbed by either compound, functions PT and PQ_{ROS} = 1):

$$\frac{d[O_2^{\cdot -}]}{dt} = PQ_{ROS} * \frac{v4}{\left(1 + \frac{[O_2^{\cdot -}]}{Ki}\right)} * \left(\frac{EP_{faulty}}{EP_{faulty} + EP_{functional}}\right) - (k1[MnSOD] + k2 + k3 * PT)[O_2^{\cdot -}]$$

$$\frac{d[H_2O_2]}{dt} = \left(\frac{1}{2}k1[MnSOD] + k2\right)[O_2^{\cdot -}] - k5[H_2O_2] * SKN1\_activity\ (GST)$$

$$\frac{d[EP_{usable}]}{dt} = v8(\mathbf{mtDNA})\left(1 - \left[\frac{[mATFS1]^n}{K_b{}^n + [mATFS1]^n}\right]\right) + k9[EP_{faulty}] * Kc([HSP60] + [HSP70]) - k10[EP_{usable}] * Kros[[O_2^{\cdot -}] + [H_2O_2]] - k11[EP_{usable}] * Kc([HSP60] + [HSP70])$$

$$\frac{d[EP_{functional}]}{dt} = k11[EP_{usable}] * Kc([HSP60] + [HSP70]) - k12[EP_{functional}] * Kp([mAAA] + [iAAA])$$

$$\frac{d[EP_{faulty}]}{dt} = v13(\Delta\mathbf{mtDNA})\left(1 - \left[\frac{[mATFS1]^n}{K_b{}^n + [mATFS1]^n}\right]\right) + k10[EP_{usable}] * Kros[[O_2^{\cdot -}] + [H_2O_2]] - k9[EP_{faulty}] * Kc([HSP60] + [HSP70]) - k14[EP_{faulty}] * Kp([mAAA] + [iAAA])$$

***Rate-dependent discrete events for changes in mtDNA copies:**

Damage to mitochondrial DNA is assessed here in totality. All perturbations (mainly point mutations and deletions) are regarded as a change in mitochondria DNA that will lead to dysfunctional protein synthesis and deleterious faulty protein aggregation. Copy numbers vary from mitochondrion to mitochondrion (2–10 copies), but it is assumed here that the mean copy number is 6, and the distribution falls rapidly in either direction. It is also assumed here that all mitochondria begin with ~15% perturbed mitochondrial DNA (~1 copy per mitochondrion), and that all subsequent DNA damage is both rate-dependent and ROS-dependent [16]. The damage rate is also not always the same for every mitochondrion, and therefore is represented by a stochastic ROS-dependent rate equation **(Eq. 1)** with a mean number of events per week and a corresponding exponential distribution for the initial trigger and recurrence time. Additionally, once mtDNA has been altered, it is apparent that the cell works to remove it when unstressed [3]; however, following the stressor-mediated nuclear activation of ATFS-1, ΔmtDNA is more easily sustained [17] **(Eq. 2)**.

$$(\mathbf{1.})\ Mean\ number\ of\ mtDNA\ altering\ events\ per\ week = damageRate * ([O_2^{\cdot -}] + [H_2O_2])$$

$$(\mathbf{2.})\ Mean\ number\ of\ \Delta mtDNA\ removal\ events\ per\ month = destroyRate * \left(1 - \left[\frac{[nATFS1]^n}{K_b{}^n + [nATFS1]^n}\right]\right)$$

***Dynamic expressions displayed in blue:**

Concentrations of proteases, chaperone proteins, ETC assembly factors, and superoxide dismutase are determined by total cellular concentrations following nuclear synthesis (as outlined in the cell-level system-dynamics methods). Additionally, the inhibitory factor governing the repression of mtDNA-mediated OXPHOS protein production is determined by the total mitochondrial concentration of ATFS-1 (all mitochondria presumably contain the same concentration at any given time due to the nature of the global response over a cell).

Box S2 (continued): Equations and event statements pertaining to mitochondrion-level biochemical kinetics.

Differential equations governing the time-dependent amounts of key substrates and variables involved in energy metabolism, condensed from simplified models of mitochondrial ATP production [50, 51] and expanded to include synthesis and breakdown of NAD^+ as well as NAD^+-dependent deacetylase (sirtuin) activity [26, 52–54]. The mathematics employed here were developed using some logistical expressions, converted parameters and relative substrate abundances from Saa and Siqueira (2013), however, our current model breaks free from previous constructs that assume constant total adenine and adenosine nucleotide availabilities. The current adaptations of the model relate to the age-dependent OXPHOS complex integrity as well as dynamically changing nicotinamide/adenine nucleotide and ADP substrate availabilities. Expressions were best translated to be used in our system dynamics interface, with the critical flux rates outlined below:

$$\frac{d[NucleotidePool]}{dt} = 5.0\frac{mM}{min} - 20.0\frac{mM}{min}\left(\frac{[NucleotidePool]}{[NucleotidePool] + 50\ mM}\right) + \frac{0.5}{min}[NMN + AMN] - 0.0075[NucleotidePool]$$

$$\frac{d[NAD^+]}{dt} = 20.0\frac{mM}{min}\left(\frac{[NucleotidePool]}{[NucleotidePool] + 50\ mM}\right) - \frac{3.0}{min}[NAD^+] + \left[K_{NADH} * [NADH] * \left(\frac{EP_{functional}}{EP_{functional} + EP_{faulty}}\right)\right] - \left\{6.2\frac{mM}{min}\left(\frac{[NAD^+]}{[NAD^+] + K_{NAD+}}\right)\right\}$$

$$\frac{d[NADH]}{dt} = \frac{3.0}{min}[NAD^+] - \left[K_{NADH} * [NADH] * \left(\frac{EP_{functional}}{EP_{functional} + EP_{faulty}}\right)\right] - \frac{0.5}{min}[NADH]$$

$$\frac{d[NMN + AMN]}{dt} = \frac{2\ Mononucleotides}{1\ Dinucleotide}\left\{6.2\frac{mM}{min}\left(\frac{[NAD^+]}{[NAD^+] + K_{NAD+}}\right)\right\} - \left\{\left(\frac{0.5}{min} + \frac{2.0}{min}\right)[NMN + AMN]\right\}$$

$$\frac{d[\psi]}{dt} = \frac{8.6\ mV}{mM}\left[K_{NADH} * [NADH] * \left(\frac{EP_{functional}}{EP_{functional} + EP_{faulty}}\right)\right]$$

$$\frac{d[P_i]}{dt} \approx 0 \approx SS\ \therefore\ [P_i] = uniform(20, 25)\ mM$$

$$\frac{d[ADP]}{dt} = v6 - \frac{0.1}{min}[ADP] - \frac{3.2}{min}[ADP]\left(\frac{EP_{functional}}{EP_{functional}+EP_{faulty}}\right) * \left(\frac{[P_i]}{[P_i]+10\ mM}\right) * \frac{\psi^{2.5}}{\psi^{2.5}+K_\psi} + 2.0\frac{mM}{min} * \left(\frac{[ATP]}{[ATP]+6.0\ mM}\right)$$

$$\frac{d[ATP]}{dt} = \frac{3.2}{min}[ADP]\left(\frac{EP_{functional}}{EP_{functional}+EP_{faulty}}\right) * \left(\frac{[P_i]}{[P_i]+10\ mM}\right) * \frac{\psi^{2.5}}{\psi^{2.5}+K_\psi} - 2.0\frac{mM}{min} * \left(\frac{[ATP]}{[ATP]+6.0\ mM}\right) - k7[ATP]$$

$$K_{NADH} = \frac{7.0}{min}\ ;\ K_{NAD+} = 0.01\ mM\ ;\ K_\psi = 131\ mV$$

Oxygen consumption (J_O), as dictated by both OXPHOS activity and ROS production (pmol/min/worm):

$$\frac{d[J_O]}{dt} = \frac{3.5\ L * pmol}{worm * mmol}\left(\left[\frac{1}{4}K_{NADH} * [NADH] * \left(\frac{EP_{functional}}{EP_{functional}+EP_{faulty}}\right)\right] + \frac{1\ mM}{1000000\ nM}\left[\boldsymbol{PQ_{ROS}} * \frac{v4}{\left(1+\frac{[O_2^{\cdot -}]}{Ki}\right)} * \left(\frac{EP_{faulty}}{EP_{functional}+EP_{faulty}}\right)\right]\right)$$

Sirtuin activity, as dictated by both NAD^+ breakdown and stilbenoid polyphenol exposure:

$$\frac{d[Sirtuin\_activity]}{dt} = \boldsymbol{PT} * \left(\frac{[NAD^+]}{[NAD^+] + K_{NAD+}}\right)$$

Box S3: Conditional and rate-dependent statements governing the rule-based categorization of mitochondrial stress, via the agent-based paradigm.

It is assumed that all mitochondria in the early stages of life are initially fully functioning and unstressed [18, 19]. As life progresses, the mitochondria undergo various levels of stress [19, 20], which have been roughly phenotyped and categorized for the purposes of the agent-based model. In our model, a mitochondrion can be **(A.)** functioning and optimal capacity (maximal ATP production), with little to no available stressors (ROS, accumulated proteins, damaged DNA), **(B.)** functioning below maximum capacity but still within the optimal range, in the presence of increased stressor concentrations, **(C.)** significantly stressed and malfunctioning with respect to energy production, or **(D.)** severely stressed with high amount of damaged genetic material, thus producing very little ATP and large amounts of superoxide. The transitions between these states are rate-dependent and governed by the corresponding stressors that account for these pathological micro-phenotypes [19, 20], as seen in the equations below. The constants involved in facilitating these stress and recovery transitions have been optimized (*StressConstant = 0.850 events* $\%EP_{faulty}^{-1}$ nM_{ROS}^{-1} hr^{-1}; *RecoveryConstant = 1.775 events* $\%EP_{faulty}$ nM_{ROS} hr^{-1}) so that the stress-state results relate closely to true biological stressor levels in WT *C. elegans* [3, 17]. Relative to these stress-states, the model accounts for mitochondrial autophagy (mitophagy), where the cell will recognize the existence of damaged mitochondria and effectively destroy such organelles in a rate-dependent manner following retrieval of such stress signals [21, 22]. The stress signals emitted from the mitochondria vary in magnitude from state to state, as defined by the model—where insignificantly-stressed mitochondria will weakly activate the mitophagic mechanisms, significantly stressed mitochondria will most effectively activate mitophagy, and severely stressed mitochondria will suffer from hypothesized UPR^{mt}-mediated overprotection and compromised stress signaling. Mitophagy here is assumed to be initiated by ineffective mitochondrial protein transport as well as SKN-1 or DAF-16 activation [22, 23, 34, 55, 56], and is therefore inversely correlated with these corresponding activities (outlined in the cell-level system-dynamics methods). Mitochondrial recycling/biogenesis is also a concomitant event that occurs within mitochondrial stress and quality control in the aging *C. elegans* [22], and it is here accounted for by (1.) the movement from high stress states to low stress states, and (2.) a timeout-triggered (every 1000-3000 min) agent-addition event. Such agent addition events occur more rapidly upon SKN-1 activation [22, 55], where biogenesis is increased by 7–20% at maximum activity.

<u>Stochastic rates for movement of increasing stress states, based on protein accumulation:</u>

$$\text{Transition 1}: Mean\ events\ per\ hour = StressConstant * [EP_{faulty}] * ([O_2^{\cdot -}] + [H_2O_2])$$

$$\text{Transition 2}: Mean\ events\ per\ hour = StressConstant * [EP_{faulty}] * ([O_2^{\cdot -}] + [H_2O_2])$$

$$\text{Transition 3}: Mean\ events\ per\ hour = StressConstant * [EP_{faulty}] * ([O_2^{\cdot -}] + [H_2O_2])$$

<u>Stochastic rates for movement of decreasing stress states, based on recovery of mitochondrial quality:</u>

$$\text{Transition 4}: Mean\ events\ per\ hour = RecoveryConstant * \frac{1}{[EP_{faulty}] * ([O_2^{\cdot -}] + [H_2O_2])}$$

$$\text{Transition 5}: Mean\ events\ per\ hour = RecoveryConstant * \frac{1}{[EP_{faulty}] * ([O_2^{\cdot -}] + [H_2O_2])}$$

$$\text{Transition 6}: Mean\ events\ per\ hour = RecoveryConstant * \frac{1}{[EP_{faulty}] * ([O_2^{\cdot -}] + [H_2O_2])}$$

<u>Mito-transport-, SKN-1-, and DAF-16-dependent [34] rates of mitochondrial loss via mitophagy (Segments "R" and "B" represent xenobiotic-induced perturbations of mitophagy rate, outlined in the last section. When unperturbed, R = 1 and B = 1):</u>

$$\text{Transition 7}: Mean\ events\ per\ day = 10\% \left(1 - \left(\frac{[TIMM23]}{[TIMM23] + Kt}\right)\right) * \mathbf{R} * \mathbf{B} * \text{DAF16\_activity} * \text{SKN1\_activity}$$

$$\text{Transition 8}: Mean\ events\ per\ day = 95\% \left(1 - \left(\frac{[TIMM23]}{[TIMM23] + Kt}\right)\right) * \mathbf{R} * \mathbf{B} * \text{DAF16\_activity} * \text{SKN1\_activity}$$

$$\text{Transition 9}: Mean\ events\ per\ day = 30\% \left(1 - \left(\frac{[TIMM23]}{[TIMM23] + Kt}\right)\right) * \mathbf{R} * \mathbf{B} * \text{DAF16\_activity} * \text{SKN1\_activity}$$

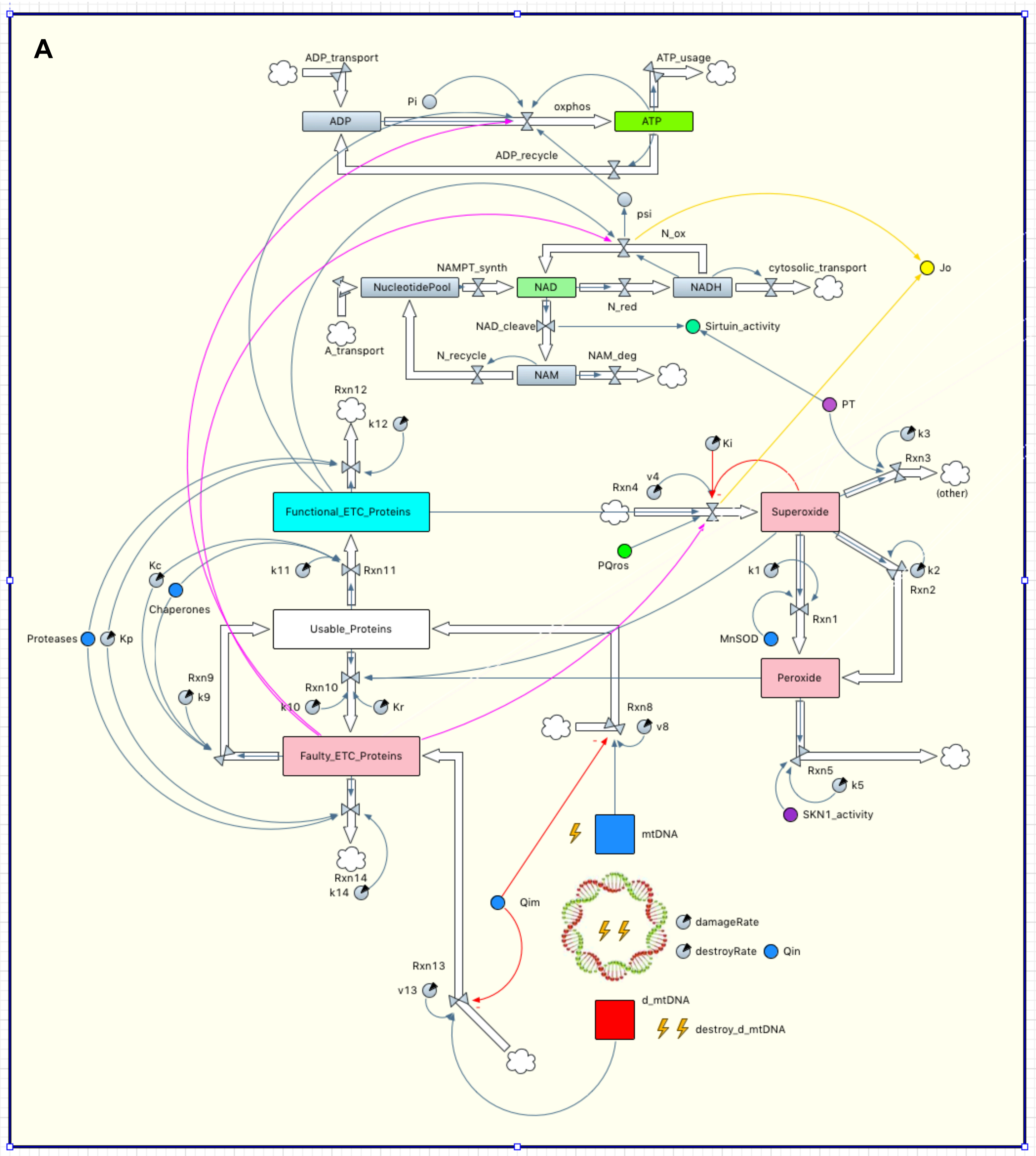

Figure S1A. Schematics of mitochondrion-level modeling dynamics:
mitochondrion-level biochemical SD network in AnyLogic.

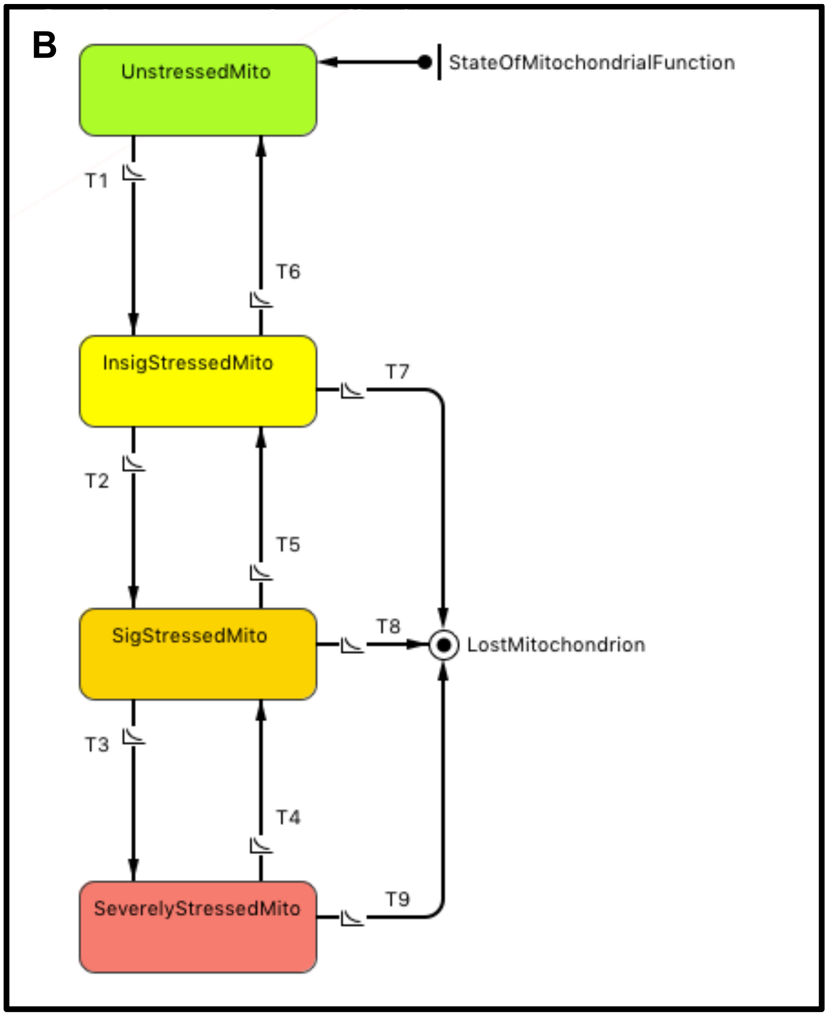

Figure S1B. Schematics of mitochondrion-level modeling dynamics: state-chart delineating the categorization of mitochondrial stress within the agent-based aging paradigm.

Table S3. Parameters of interest for the proposed intracellular biochemical system.

Parameter	Symbol	Value Range	Units	Source
Zero-order production constant for ATFS-1	*v15*	8×10^{-2}	% min^{-1}	100%[SS]
Maximum UPR^{mt}-mediated production of ATFS-1	*v15max*	v15 (1.5)	% min^{-1}	[18,24][A]
Hill coefficient for graded UPR^{mt}-mediated response	*n*	1 – 2	unitless	[18][A]
ATFS-1 DNA-binding affinity constant	*Kb*	38.03	%	[3, 18][O]
Rate constant for mitochondrial import of ATFS-1	*Kmt*	95.01	min^{-1}	[18][O]
EC_{50} for ATP-dependent mitochondrial transport efficacy, presumably governed by Michaelian kinetics	*Ka*	11.95	mM	[3, 18][O]
Rate constant for nuclear import of ATFS-1	*Kni*	5.00	min^{-1}	[18][O]
Elimination constant for ATFS-1	*Ke15*	8.0×10^{-4}	min^{-1}	[15, 18][A]
Zero-order production constant for TIMM23	*Ko16*	1.4×10^{-3}	% min^{-1}	100%[SS]
Maximum UPR^{mt}-mediated production of TIMM23	*Kmax16*	Ko16 (0.7 – 1.5)	%	[18][A]
Elimination constant for TIMM23	*Ke16*	3.5×10^{-5}	min^{-1}	[15][C]
EC_{50} for TIMM23-dependent mitochondrial transport efficacy, presumably governed by Michaelian kinetics	*Kt*	34.843	%	[3, 25][O]
Zero-order production constant for HSP60	*Ko17*	1.9×10^{-2}	% min^{-1}	100%[SS]
Maximum UPR^{mt}-mediated production of HSP60	*Kmax17*	Ko17 (1.0)	%	[18,24][A]
Elimination constant for HSP60	*Ke17*	1.9×10^{-4}	min^{-1}	[15][C]
Zero-order production constant for HSP70	*Ko18*	1.9×10^{-2}	% min^{-1}	100%[SS]
Maximum UPR^{mt}-mediated production of HSP70	*Kmax18*	Ko18 (1.7 – 3.0)	%	[18,24][A]
Elimination constant for HSP70	*Ke18*	1.9×10^{-4}	min^{-1}	[15][C]
Zero-order production constant for i-AAA	*Ko19*	2.6×10^{-2}	% min^{-1}	100%[SS]
Maximum UPR^{mt}-mediated production of i-AAA	*Kmax19*	Ko19 (1.0)	%	[24][A]
Elimination constant for i-AAA	*Ke19*	2.6×10^{-4}	min^{-1}	[15][C]
Zero-order production constant for m-AAA	*Ko20*	2.6×10^{-2}	% min^{-1}	100%[SS]
Maximum UPR^{mt}-mediated production of m-AAA	*Kmax20*	Ko20 (0)	%	[24][A]
Elimination constant for m-AAA	*Ke20*	2.6×10^{-4}	min^{-1}	[15][C]
Zero-order production constant for MnSOD	*Ko21*	3.0×10^{-6}	mM min^{-1}	[3,5][SS]
Maximum UPR^{mt}-mediated production of MnSOD (Indirect)	*Kmax21*	Ko21 (0.5 – 1.5)	mM min^{-1}	[26][A]
Elimination constant for MnSOD	*Ke21*	$6.0 – 12.0 \times 10^{-4}$	min^{-1}	[27][A]
Zero-order constant for glycolysis-mediated ATP production	*v22*	0 – 0.18	mM min^{-1}	[28][A]
Maximum UPR^{mt}-mediated production of glycolysis factors	*v22max*	v22 (0.5 – 3.0)	mM min^{-1}	[18,24,29][A]
Cytosolic ATP turnover	*k23*	2.1	min^{-1}	[8][C]

[SS] Optimized in the simulation to achieve the reported unstressed physiological steady-state value, percentage or range.
[O] Individually optimized value, generated using AnyLogic calibration experiments to best fit or emulate reported kinetic curves.
[A] Quantitated assumption based on qualitative evidence.
[C] Units converted to adhere to model standards.

Box S4: Equations pertaining to cell-level biochemical kinetics.

Dynamics of ATFS-1 concentrations within the cytosolic, mitochondrial, and nuclear compartments (PQ_{UPRmt} is a function of paraquat exposure, outlined in the last section):

$$\frac{d[ATFS1]}{dt} = v_{15} + v15_{max}\left(\frac{[nATFS1]^n}{{K_b}^n + [nATFS1]^n}\right) - K_{mi}\left(\frac{[ATP]_{avg}}{[ATP]_{avg} + Ka}\right)\left(\frac{[TIMM23]}{[TIMM23] + Kt}\right)[ATFS1] - K_{ni}[ATFS1]$$

$$\frac{d[mATFS1]}{dt} = \boldsymbol{PQ_{UPRmt}} * K_{mi}\left(\frac{[ATP]_{avg}}{[ATP]_{avg} + Ka}\right)\left(\frac{[TIMM23]}{[TIMM23] + Kt}\right)[ATFS1] - K_{e15}[mATFS1]$$

$$\frac{d[nATFS1]}{dt} = K_{ni}[ATFS1] * (Sirtuin\_activity) - K_{e15}[nATFS1]$$

$$\frac{d[ATFS1\_activity]}{dt} = \left[\frac{[nATFS1]^n}{{K_b}^n + [nATFS1]^n}\right]$$

*where *$[ATP]_{avg}$* represents the average ATP concentration surveyed over a population of mitochondria for a given cell.

Dynamics and normalized activity of DAF-16 and SKN-1 transcription factors, controlled by oxidative stress signaling (and to a degree sirtuin-mediated deacetylation), which leads to accumulation in the nuclear compartment:

$$\frac{d[nDAF\_16]}{dt} = K_{dni} * \left(\frac{[ROS]_{avg}}{[ROS]_{avg} + K_{dROSact}}\right) * (K_{dSIRact} * Sirtuin\_activity) - K_{de}[nDAF\_16]$$

$$K_{dni} = 1.0\frac{\%}{min} \;\wedge\; K_{de} = \frac{0.01}{min} \;\wedge\; K_{dROSact} = 100\ nM \;\wedge\; K_{dSIRact} = 75\%$$

$$\frac{d[DAF16\_activity]}{dt} = 1.0 + \left(\frac{[nDAF\_16]^2}{50\%^2 + [nDAF\_16]^2}\right)$$

$$\frac{d[nSKN\_1]}{dt} = K_{sni} * \left(\frac{[ROS]_{avg}}{[ROS]_{avg} + K_{sROSact}}\right) * (K_{sSIRact} * Sirtuin\_activity) - K_{se}[nSKN\_1]$$

$$K_{sni} = 1.0\frac{\%}{min} \;\wedge\; K_{se} = \frac{0.01}{min} \;\wedge\; K_{sROSact} = 75\ nM \;\wedge\; K_{sSIRact} = 25\%$$

$$\frac{d[SKN1\_activity]}{dt} = 1.0 + \left(\frac{[nSKN\_1]^2}{50\%^2 + [nSKN\_1]^2}\right)$$

*where *$[ROS]_{avg}$* represents the average ROS concentration surveyed over a population of mitochondria for a given cell.

Box S4 (continued): Equations pertaining to cell-level biochemical kinetics.

Simplified differential equations for time-dependent protein synthesis and degradation of key mito-proteins controlled primarily by the UPR^{mt}:

$$\frac{d[TIMM23]}{dt} = K_{o16} + K_{max16}\left(\frac{[nATFS1]^n}{{K_b}^n + [nATFS1]^n}\right) - K_{e16}[TIM23]$$

$$\frac{d[HSP60]}{dt} = K_{o18} + \left(\frac{[TIMM23]}{[TIMM23] + Kt}\right)\left(K_{max18}\left(\frac{[nATFS1]^n}{{K_b}^n + [nATFS1]^n}\right)\right) - K_{e18}[HSP60]$$

$$\frac{d[HSP70]}{dt} = K_{o19} + \left(\frac{[TIMM23]}{[TIMM23] + Kt}\right)\left(K_{max19}\left(\frac{[nATFS1]^n}{{K_b}^n + [nATFS1]^n}\right)\right) - K_{e19}[HSP70]$$

$$\frac{d[iAAA]}{dt} = K_{o20} + \left(\frac{[TIMM23]}{[TIMM23] + Kt}\right)\left(K_{max20}\left(\frac{[nATFS1]^n}{{K_b}^n + [nATFS1]^n}\right)\right) - K_{e20}[iAAA]$$

$$\frac{d[mAAA]}{dt} = K_{o21} + \left(\frac{[TIMM23]}{[TIMM23] + Kt}\right)\left(K_{max21}\left(\frac{[nATFS1]^n}{{K_b}^n + [nATFS1]^n}\right)\right) - K_{e21}[mAAA]$$

$$\frac{d[MnSOD]}{dt} = K_{o22} + \left(\frac{[TIMM23]}{[TIMM23] + Kt}\right)\left(K_{max22}\left(\frac{[nATFS1]^n}{{K_b}^n + [nATFS1]^n}\right) * (DAF16\_activity)\right) - K_{e22}[MnSOD]$$

*DAF16_activity is coupled with MnSOD production as a known antioxidant response element for this transcription factor. All mitochondrial proteins are also reliant on the efficacy of their transport systems, as delineated by the mathematical expression that utilizes the translocase abundance.

Glycolysis-mediated ATP production, upregulated upon UPR^{mt} activation:

$$\frac{d[ATP]}{dt} = v24 + v24max\left(\frac{[nATFS1]^n}{{K_b}^n + [nATFS1]^n}\right) - k25[ATP]$$

Box S5: Conditional and rate-dependent statements governing the rule-based categorization of cell integrity, dependent upon the mitochondrial agent population within each cell agent.

Respiratory capacity is reportedly lost when deleterious mtDNA mutations hit a threshold value between 60% and 90% [17, 21, 29]. It is assumed that this threshold value can be translated directly as general loss of mitochondrial function, which have been outlined by the conditional eustress and distress states of the model. This distribution of threshold values (triangular distribution used in the model: 60%-62.5%-75%) is used to govern the loss of cellular integrity and the increased probability in cell death. Recovery of such cells in the model is possible, if their mitochondria can restore enough function to return below the lowest possible threshold value of 60%. This strict assumption is made to uphold the standards of true biological systems, which face a great deal of difficulty restoring homeostasis when mitochondrial populations are largely compromised [30]. It has been reported that humans begin to lose brain volume around the age of 40, at a rate of 5% every decade, mainly due to spontaneous neuronal degeneration [31]. If we assume the average human life expectancy at birth is 70 years and if we assume these degradation checkpoints are similarly conserved among eukaryotes like *C. elegans* (average N2 lifespan @ 25 ^{O}C: 20 days [32]), then neuronal death will begin to occur within the nematode around day 11.5 at a rate of 5% every 2.8 days, with a more rapid decline immediately before death. That being said, it has been reported that *C. elegans* do not lose substantial neuronal integrity [57], yet vacuolated compromised neuronal cells and neurodegenerative responses have been observed in these organisms [58]. In light of these findings, the model was designed to primarily determine the dynamics of the age-compromised state for energetically-demanding cells, while attempting to shed light on potential tissue degeneration thereafter. Once mitochondrial conditions force the cell into a compromised state, they have a limited time to recover or they are destroyed. This rate is defined in the model by an exponential distribution that has been characterized meet expected physiological neuronal lifespan.

Stochastic Condition defining loss of general mitochondrial integrity:

$$\left(\frac{DefectiveMitochondria}{TotalMitoCount}\right) > \left(triangular(60\%, 62.5\%, 75\%)\right)$$

Static condition defining cell recovery based on restoration of general mitochondrial quality:

$$\left(\frac{DefectiveMitochondria}{TotalMitoCount}\right) < 60\%$$

Stochastic rate of cell death *subsequent* to loss of mitochondrial function:

$$Mean\ number\ of\ compromised\ cell\ death\ events\ per\ day = 8.25 \times 10^{-2}$$

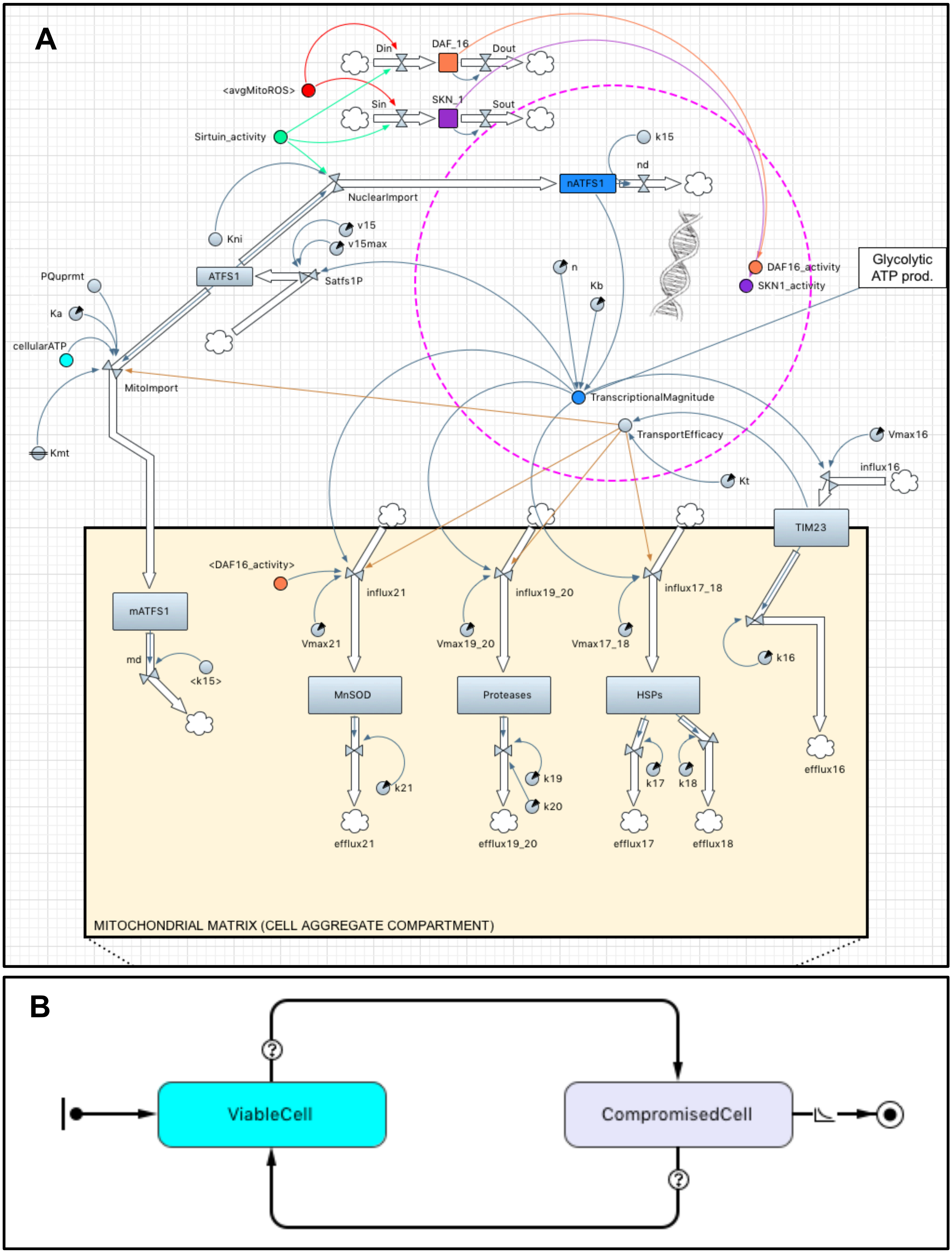

Figure S2. Schematics of cell-level modeling dynamics. (A) Cell-level biochemical SD network in AnyLogic. (B) State-chart for the conditional categorization of each cell's mitochondrial integrity and cell death.

Computational methods for organism-level biomarker and endpoint capture

Box S6: Equations used to capture tissue concentrations of parameters of interest, where "i" is the current number of total mitochondria in the system and "n" is the current number of neurons. The system initially contains 65 neurons for simplicity.

$$Mitochondrial\ count\ at\ time\ t = i(t)$$

$$Initial\ cell\ count = n_o \quad ; \quad Current\ cell\ count\ at\ time\ t = n(t)$$

$$Percent\ cell\ mortality = 100\% \left(\frac{n_o - n(t)}{n_o}\right)$$

$$Average\ tissue\ mtROS\ concentration = \left(\frac{\sum_0^i [Peroxide + Superoxide]}{i(t)}\right)$$

$$Average\ tissue\ ATP\ concentration = \left(\frac{\sum_0^i [ATP]}{i(t)} + \frac{\sum_0^n [ATP_{Glycolysis}]}{n(t)}\right)$$

$$Percent\ mtDNA\ copies\ damaged = \left(\frac{\sum_0^i [\Delta mtDNA]}{\sum_0^i [mtDNA] + \sum_0^i [\Delta mtDNA]}\right) * 100\%$$

$$Average\ tissue\ NAD^+\ concentration = \left(\frac{\sum_0^i [NAD^+]}{i(t)}\right)$$ → Normalized to earliest time point (relative)

$$Average\ tissue\ oxygen\ consumption\ rate = \left(\frac{\sum_0^i [J_O]}{i(t)}\right)$$

Modeling alterations for virtual pharmacological and genetic perturbations

Use of Rapamycin and Bafilomycin

One virtual pharmacological perturbation mode within the model addresses mitophagy flux. One perturbation deals with the established role of the mechanistic target of rapamycin (mTOR), which normally acts to inhibit selective mitophagy and reduce the rate of mitochondrial degradation. It has been identified that this protein is well conserved among eukaryotes, and such a homolog of this protein has been identified in *C. elegans* [33] which is indeed, as it is in mammals, inhibited upon rapamycin administration [34]. It has been well established that rapamycin acts through this mechanism to decrease heteroplasmic mtDNA accumulation in immortalized neurons [35] and to alleviate general mitochondrial dysfunction in whole rat brains (as assessed by oxidative damage biomarkers and ATP levels) [36]. Assuming this therapeutic effect is mechanistically conserved among eukaryotes, rapamycin is used here as a virtual xenobiotic to increase the rate of mitophagy. It is administered virtually within different dosing schemes at a concentration of 15 nM (which equates to 18.3 μg/L, within the therapeutic range for serum levels of 5-20 μg/L [35]). Once administered instantaneously, it virtually affects the cells by a maximal fraction (X_R) of 1.5 in relation to its EC_{50} (estimated at ~10 nM [35]), and it begins to decrease in concentration with an aqueous half-life of about 10 h [37].

Bafilomycin A1, in contrast, facilitates an inhibition of autophagy in *C. elegans* by preventing the maturation and accumulation of autophagic vesicles [38]. Bafilomycin A1 is virtually administered here within different dosing schemes in a similar fashion at 10 nM, with a maximal fraction (X_B) of 0.5, an EC_{50} of ~7 nM [38], and a low-end estimate of the culture half-life at 40 h (adapted from *in vivo* pharmacokinetic data for the structurally similar azithromycin [39]). The pharmacodynamic equations outlined below (Box S7) for rapamycin or bafilomycin administration are multiplied by the stochastic mitophagy transitions 7, 8 and 9 within the mitochondrial agent-based paradigm (Box S3), and subsequently integrated over time for different dosing schemes (*note: there is no direct effect when concentrations of either chemical are zero*).

Use of Paraquat

As an additional and different type of mechanistic perturbation, paraquat is virtually used here for its aging-related pro-oxidant properties within the mitochondria [40–42] and for its ability to induce the UPR^{mt} [18]. Paraquat is virtually administered here in a similar fashion to rapamycin and bafilomycin, at a concentration of 100 μM (high) or 5 μM (low), with an environmental half-life that far exceeds the normal nematode lifespan [43], and so paraquat concentrations will simply be clamped within different dosing schemes. Paraquat perturbs the system using the same types of equations, however, since paraquat perturbs the system in two different areas (superoxide production rate *and* mitochondrial import of ATFS-1) in two distinctly different and opposing ways, the functions for each are separately categorized, as outlined below (Box S7). For the function pertaining to its pro-oxidant properties: the constant for maximal rate increase, X_{PQR}, is set at 0.4, and the EC_{50} for this effect is set at 25 μM [estimations; 44, 45]. For the function pertaining to its UPR^{mt} activation (presumably due to lack of ATFS-1 mitochondrial import efficiency): the constant for maximal impedance effect, X_{PQU}, is set at 0.75, and the EC_{50} for this effect is set at 50 μM [estimations; 18]. These functions PQ_{ROS} and PQ_{UPRmt} (Box S7) are integrated over time for various dosing schemes and are then multiplied to the rates of mitochondrial superoxide production (Box S2) and mitochondrial ATFS-1 import (Box S4), respectively (*note: there is no direct effect when exposure concentration of paraquat is zero*).

Use of Pterostilbene

Plant-derived stilbenes have been extensively studied as anti-aging therapies, with special emphasis on resveratrol and pterostilbene (prominent constituents of blueberries, red grapes and red wine products). Pterostilbene has been recorded as a more potent neuromodulator than resveratrol in aging, even when administered at low doses [46], and therefore is used as the stilbene in this simulation study. Pterostilbene has direct radical scavenging properties and mitigates ROS through highly conserved oxidative stress response pathways as well, via sirtuin activity that acts upon transcription factors such as SKN-1 and DAF-16 in worms [47, 52]. These effects ultimately manifest as reduced ROS content and increased bioenergetic efficiency in aging organisms, and specifically has reduced the levels of ROS in *C. elegans* by about 26% when constantly administered at a concentration of 100 μM [47]. Because of these observations, the pterostilbene function used in this model (same format as rapamycin) utilizes an EC_{50} of 20–60 μM (conservative estimate) and a maximal ROS-mitigating ($X_{PTR)}$ and sirtuin-activating effect (X_{PTS}) of 0.26 and 0.5, respectively, which are applied to the non-peroxide-forming superoxide elimination rate and the Sirtuin_activity dynamic variable. Pterostilbene is administered here at a dose of 100 μM and assumes a culture half-life of 12 h due to its poor aqueous stability [48] and rapid pharmacokinetic clearance [49]. All of these factors are incorporated into the function *PT* (Box S7), which is applied to the rates (seen in Box S2) of mitochondrial superoxide elimination and peroxide elimination (*note: there is no direct effect when exposure concentration of pterostilbene is zero*).

Varied Dosing Schemes

Dosing schemes for rapamycin, bafilomycin, pterostilbene and paraquat were altered and/or combined to produce an array of exposure scenarios. For rapamycin, bafilomycin and pterostilbene, doses were administered at 0 min and every 2500 min thereafter; the resulting cellular exposure at any given time point was determined by their culture half-life kinetics. For paraquat, high or low doses were clamped to maintain a constant exposure value. For all of these xenobiotics, their administration was performed constantly, early (0–10000 min), mid-life (10000–15000 min), or late (15000–30000 min).

Genetic Perturbations

To identify the role of key genes as they relate to this system, several were virtually knocked down and the endpoints of interest were observed and compared to the control group. Such genes were those encoding for MnSOD, SKN-1, and DAF-16. To make these virtual perturbations, the rate of productive expression for these functional proteins were fractionalized for the duration of the simulation, to simulate such reduced genetic accessibility for the corresponding genes.

Box S7: Functions used to define virtual pharmacological perturbations within the model.

$$\text{Function of pharmacological perturbation by rapamycin: } \boldsymbol{R} = 1 + X_R\left(\frac{[Rapamycin]}{[Rapamycin] + EC_{50}}\right)$$

$$\text{Function of pharmacological perturbation by bafilomycin: } \boldsymbol{B} = 1 - X_B\left(\frac{[Bafilomycin]}{[Bafilomycin] + EC_{50}}\right)$$

$$\text{Function of increased ROS production by paraquat: } \boldsymbol{PQ_{ROS}} = 1 + X_{PQR}\left(\frac{[Paraquat]}{[Paraquat] + EC_{50}}\right)$$

$$\text{Function of ATFS-1-mito-import inhibition by paraquat: } \boldsymbol{PQ_{UPRmt}} = 1 - X_{PQU}\left(\frac{[Paraquat]}{[Paraquat] + EC_{50}}\right)$$

$$\text{Function of mito-sirtuin activation by pterostilbene: } \boldsymbol{PT} = 1 + X_{PTS}\left(\frac{[Pterostilbene]}{[Pterostilbene] + EC_{50}}\right)$$

$$\text{Function of radical-scavenging capacity by pterostilbene: } \boldsymbol{PT} = 1 + X_{PTR}\left(\frac{[Pterostilbene]}{[Pterostilbene] + EC_{50}}\right)$$

Global Sensitivity Analysis

Table S4. Normalized sensitivity coefficients (SC) reported for age-dependent mitochondrial population numbers, ROS accumulation, and percent mtDNA heteroplasmy at early (3000 min), midlife (10,000 min) and late (22,000 min) time points throughout the simulation (parameter deemed to have sensitive control when |SC| ≥ 1). Parameters that elicited significant changes to specific response variables are bolded. The normalized sensitivity coefficients also represent positive or negative changes to response variables, as indicated by the sign that precedes each value.

**Parameter**	**Response variable**								
	Mitochondrial population count			Steady-state ROS concentration			mtDNA heteroplasmy percentage		
	Early	Midlife	Late	Early	Midlife	Late	Early	Midlife	Late
k1	+0.0326	+0.3922	**+1.7021**	-0.1884	-0.0622	+0.0749	-0.6544	-0.5552	-0.1316
k2	+0.0978	+0.4392	+0.7092	-0.4384	**-1.0074**	**-1.0789**	-0.3135	**-1.0288**	-0.1741
k3	+0.0815	+0.2824	**+1.0402**	+0.7218	+0.3029	+0.3872	-0.2911	+0.0716	+0.0752
v4	+0.0978	+0.1412	**+1.4894**	-0.3051	-0.6650	**-1.0557**	-0.5524	-0.7417	-0.4431
k5	+0.0326	+0.1725	-0.2364	**+1.4054**	+0.9251	-0.1180	-0.2090	+0.8689	-0.2198
v6	+0.0489	-0.0157	-0.8511	+0.0167	-0.6347	-0.5773	+0.2252	-0.9644	-0.6175
k7	-0.2445	+0.0000	**+1.3239**	+0.0867	-0.3880	-0.6227	-0.3421	-0.3237	-0.1929
K_{NADH}	-0.0326	+0.1098	**+1.1584**	-0.0483	-0.2734	-0.9642	-0.1879	+0.0211	-0.3222
K_{Ψ}	+0.0326	-0.0627	**+1.0875**	+0.1417	-0.0431	**-1.0619**	-0.5574	+0.0694	-0.4438
K_{NAD+}	+0.0489	+0.4863	**+1.7730**	+0.0167	-0.1214	+0.2700	**-1.1173**	-0.7256	+0.8263
v8	-0.0326	+0.4549	+0.0236	-0.0633	+0.0264	-0.4707	-0.6271	-0.0416	-0.3666
k9	+0.0815	+0.1412	**+2.2695**	-0.0167	-0.4031	-0.5208	-0.3546	-0.2848	-0.4914
k10	-0.1630	+0.1412	**-1.5603**	+0.1250	-0.3349	-0.8619	-0.3036	-0.6079	-0.9190
Kros	-0.1630	+0.1412	+0.1182	+0.1250	-0.3349	-0.7398	-0.3036	-0.6079	-0.3637
k11	-0.0326	+0.4549	+0.0236	-0.0633	+0.0264	-0.4707	-0.6271	-0.0416	-0.3666
k12	-0.0326	+0.4549	+0.0236	-0.0633	+0.0264	-0.4707	-0.6271	-0.0416	-0.3666
Kc	-0.0489	+0.2353	+0.7565	+0.0750	-0.1488	-0.9302	**-1.1285**	+0.4009	-0.8623
Kp	+0.0000	+0.2667	**+2.2695**	-0.4468	**-1.8395**	-0.8127	-0.0697	**-1.3180**	+0.1621
v13	-0.0326	+0.4549	+0.0236	-0.0633	+0.0264	-0.4707	-0.6271	-0.0416	-0.3666
k14	-0.2282	-0.2980	+0.1891	+0.3501	-0.5980	-0.5832	-0.4852	-0.5774	-0.2580
damageRate	-0.0163	+0.1569	+0.1418	+0.2217	-0.4593	-0.4791	**-1.3910**	**-1.0532**	-0.4794
destroyRate	+0.0652	+0.2667	+0.1891	+0.0583	+0.0370	-0.6627	+0.0361	+0.6651	-0.4668
stressConstant	-0.0326	+0.1098	-0.0946	+0.0550	-0.6159	**-1.0147**	-0.5375	**-1.0504**	-0.5832
recovConstant	+0.0000	+0.1412	**+1.3712**	-0.0367	-0.1571	-0.6270	+0.0012	+0.0233	-0.2532
Kb	+0.0163	+0.0784	+0.3310	-0.2884	-0.1350	-0.8480	+0.1294	-0.0816	-0.2509
Kmt	+0.0815	-0.0941	+0.7092	+0.0117	-0.2027	-0.5101	+0.2774	+0.0539	+0.0451
Ka	-0.0489	+0.0000	+0.8747	+0.1634	+0.1755	-0.7183	-0.5860	-0.2854	-0.2989
Kni	+0.0163	+0.1098	+0.2364	+0.1617	-0.2800	-0.5324	-0.0921	-0.1588	-0.0104
Kt	-0.0163	-0.0627	**+1.5603**	-0.0767	-0.4973	-0.2604	-0.1008	-0.7479	-0.1942
$K_{dROSact}$	+0.0652	+0.0157	-0.3546	+0.4168	-0.1309	**-1.5124**	-0.1605	-0.1643	**-1.1647**
$K_{dSIRact}$	+0.0652	+0.2667	+0.8511	+0.2534	-0.3006	-0.1683	+0.0946	-0.7751	-0.4593
$K_{sROSact}$	-0.1304	-0.2353	**+1.0402**	+0.0967	-0.7151	-0.5198	+0.0547	+0.1560	-0.3485
$K_{sSIRact}$	+0.0652	-0.3608	-0.1891	+0.2767	-0.2410	-0.1924	+0.0299	-0.3476	+0.2198

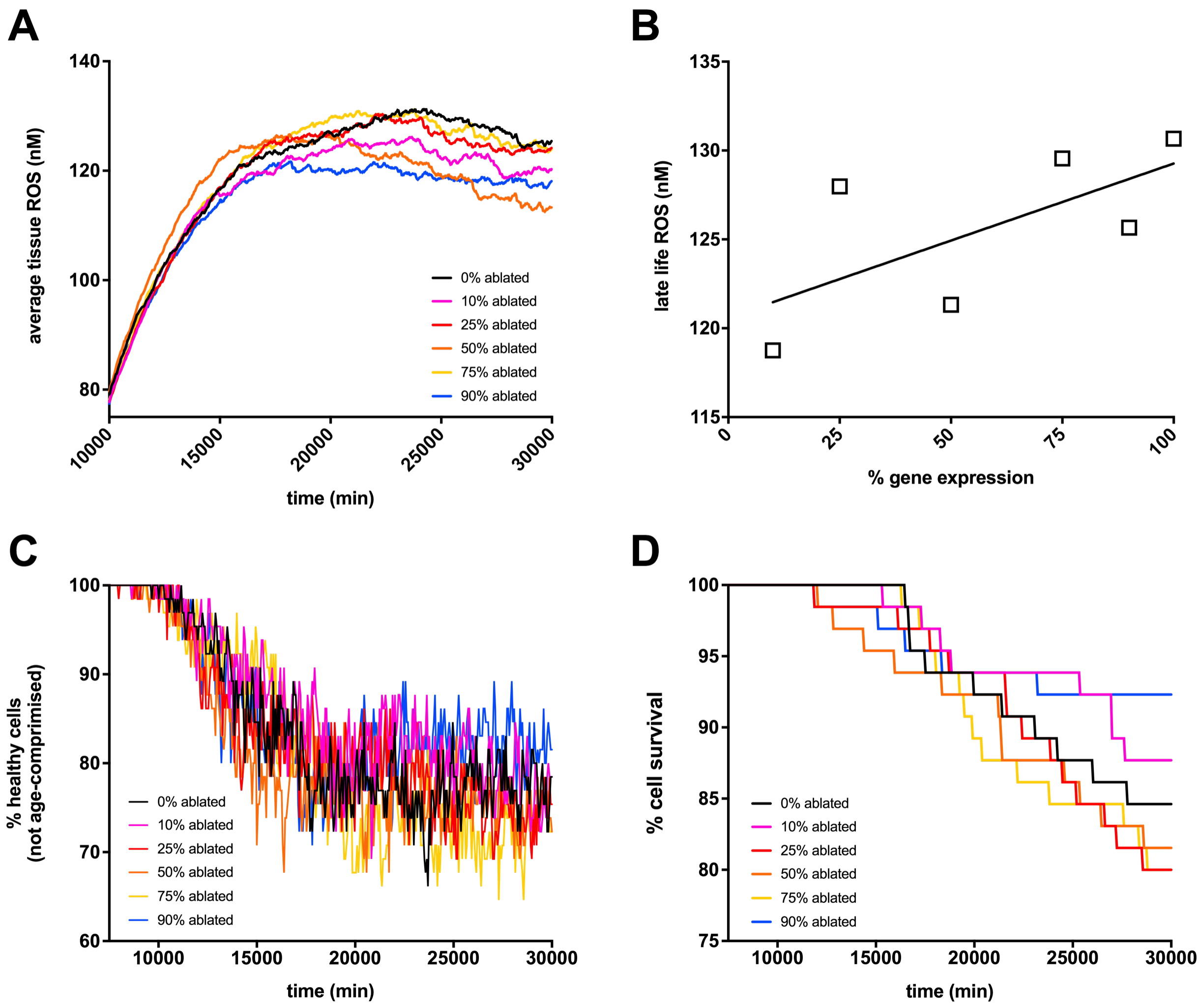

Figure S3. Varied expressional magnitude of mitochondrial manganese-dependent superoxide dismutase (MnSOD). (A) Complete survey of ROS accumulation over time after varying levels of MnSOD ablation. (B) Analysis of late life ROS output (24,000 min time point) against varying levels of MnSOD expression. (C) Viable cell survey over time after varying levels of simulated knockout. (D) Cell survival survey over time after varying levels of simulated knockout.

Supplementary References

1. McCulloch D, Gems D (2003) Body size, insulin/IGF signaling and aging in the nematode Caenorhabditis elegans. *Exp. Geront.* **38**, 129–136.
2. Wiesner RJ, Rüegg JC, Morano I (1992) Counting target molecules by exponential polymerase chain reaction: copy number of mitochondrial DNA in rat tissues. *Biochem. Biophys. Res. Commun.* **183**, 553–559.
3. Gruber J, Ng LF, Fong S, Wong YT, Koh SA, Chen C, Shui G, Cheong WF, Schaffer S, Wenk MR, Halliwell B (2011) Mitochondrial changes in ageing Caenorhabditis elegans--what do we learn from superoxide dismutase knockouts?. *PLoS One* **6**, e19444.
4. Tsang WY, Lemire BD (2002) Mitochondrial genome content is regulated during nematode development. *Biochem. Biophys. Res. Commun.* **291**, 8–16.
5. Cadenas E, Davies KJ (2000) Mitochondrial free radical generation, oxidative stress, and aging. *Free Radic. Biol. Med.* **29**, 222–230.
6. Dukan S, Farewell A, Ballesteros M, Taddei F, Radman M, Nyström T (2000) Protein oxidation in response to increased transcriptional or translational errors. *Proc. Natl. Acad. Sci. U. S. A.* **97**, 5746–5749.
7. Giorgio M, Trinei M, Migliaccio E, Pelicci PG (2007) Hydrogen peroxide: a metabolic by-product or a common mediator of ageing signals?. *Nature Rev. Mol Cell Biol.* **8**, 722–728.
8. Stewart MA, Franks-Skiba K, Chen S, Cooke R (2010) Myosin ATP turnover rate is a mechanism involved in thermogenesis in resting skeletal muscle fibers. *Proc. Natl. Acad. Sci. U. S. A.* **107**, 430–435.
9. Burd NA, Tardif N, Rooyackers O, van Loon LJ (2015) Optimizing the measurement of mitochondrial protein synthesis in human skeletal muscle. *Appl. Physiol. Nutr. Metab.* **40**, 1–9.
10. Cabiscol E, Bellí G, Tamarit J, Echave P, Herrero E, Ros J (2002) Mitochondrial Hsp60, resistance to oxidative stress, and the labile iron pool are closely connected in Saccharomyces cerevisiae. *J. Biol. Chem.* **277**, 44531–44538.
11. Chondrogianni N, Petropoulos I, Grimm S, Georgila K, Catalgol B, Friguet B, Grune T, Gonos ES (2014) Protein damage, repair and proteolysis. *Mol. Aspects Med.* **35**, 1–71.
12. Stadtman ER (2006) Protein oxidation and aging. *Free Radic. Res.* **40**, 1250–1258.
13. Sohal RS (2002) Role of oxidative stress and protein oxidation in the aging process. *Free Radic. Biol. Med.* **33**, 37–44.
14. Goto S, Radak Z (2013) Implications of oxidative damage to proteins and DNA in aging and its intervention by caloric restriction and exercise. *J. Sport Health Sci.* **2**, 75–80.
15. Nelson CJ, Li L, Jacoby RP, Millar AH (2013) Degradation rate of mitochondrial proteins in Arabidopsis thaliana cells. *J. Proteome Res.* **12**, 3449–3459.
16. Kujoth GC, Hiona A, Pugh TD, Someya S, Panzer K, Wohlgemuth SE, Hofer T, Seo AY, Sullivan R, Jobling WA, Morrow JD, Van Remmen H, Sedivy JM, Yamasoba T, Weindruch R, Leeuwenburgh C, Prolla TA (2005) Mitochondrial DNA mutations, oxidative stress, and apoptosis in mammalian aging. *Science* **309**, 481–484.
17. Lin Y, Schulz AM, Pellegrino MW, Lu Y, Shaham S, Haynes CM (2016) Maintenance and propagation of a deleterious mitochondrial genome by the mitochondrial unfolded protein response. *Nature* **533**, 416–419.
18. Nargund AM, Pellegrino MW, Fiorese CJ, Baker BM, Haynes CM (2012) Mitochondrial import efficiency of ATFS-1 regulates mitochondrial UPR activation. *Science* **337**, 587–590.
19. Hekimi S, Lapointe J, Wen Y (2011) Taking a “good” look at free radicals in the aging process. *Trends Cell Biol.* **21**, 569–576.
20. Raimundo N (2014) Mitochondrial pathology: stress signals from the energy factory. *Trends Mol. Med.* **20**, 282–292.

21. Tam ZY, Gruber J, Halliwell B, Gunawan R (2015) Context-Dependent Role of Mitochondrial Fusion-Fission in Clonal Expansion of mtDNA Mutations. *PLoS Comput. Biol.* **11**, e1004183.

22. Palikaras K, Lionaki E, Tavernarakis N (2015) Coordination of mitophagy and mitochondrial biogenesis during ageing in C. elegans. *Nature* **521**, 525–528.

23. Menzies FM, Fleming A, Rubinsztein DC (2015) Compromised autophagy and neurodegenerative diseases. *Nature Rev. Neurosci*. **16**, 345–357.

24. Nargund AM, Fiorese CJ, Pellegrino MW, Deng P, Haynes CM (2015) Mitochondrial and nuclear accumulation of the transcription factor ATFS-1 promotes OXPHOS recovery during the UPR(mt). *Mol. Cell* **58**, 123–133.

25. Ahting U, Floss T, Uez N, Schneider-Lohmar I, Becker L, Kling E, Iuso A, Bender A, de Angelis MH, Gailus-Durner V, Fuchs H, Meitinger T, Wurst W, Prokisch H, Klopstock T (2009) Neurological phenotype and reduced lifespan in heterozygous Tim23 knockout mice, the first mouse model of defective mitochondrial import. *Biochim. Biophys. Acta.* **1787**, 371–376.

26. Mouchiroud L, Houtkooper RH, Moullan N, Katsyuba E, Ryu D, Cantó C, Mottis A, Jo YS, Viswanathan M, Schoonjans K, Guarente L, Auwerx J (2013) The NAD+/sirtuin pathway modulates longevity through activation of mitochondrial UPR and FOXO signaling. *Cell* **154**, 430–441.

27. Karlsson K, Sandström J, Edlund A, Marklund SL (1994) Turnover of extracellular-superoxide dismutase in tissues. *Lab. Invest.* **70**, 705–710.

28. Bittner CX, Loaiza A, Ruminot I, Larenas V, Sotelo-Hitschfeld T, Gutiérrez R, Córdova A, Valdebenito R, Frommer WB, Barros LF (2010) High Resolution Measurement of the Glycolytic Rate. *Front. Neuroenergetics* **2**, 26.

29. Rossignol R, Faustin B, Rocher C, Malgat M, Mazat J, Letellier T (2003) Mitochondrial threshold effects. *Biochem. J.* **370**, 751–762.

30. Tower J (2015) Programmed cell death in aging. *Ageing Res. Rev.* **23**, 90–100.

31. Peters R (2006) Ageing and the brain. *Postgrad. Med. J.* **82**, 84–88.

32. Bennett CF, Wende HV, Simko M, Klum S, Barfield S, Choi H, Pineda VV, Kaeberlein M (2014) Activation of the mitochondrial unfolded protein response does not predict longevity in Caenorhabditis elegans. *Nature Commun.* **5**, 3483.

33. Long X, Spycher C, Han ZS, Rose AM, Müller F, Avruch J (2002) TOR deficiency in C. elegans causes developmental arrest and intestinal atrophy by inhibition of mRNA translation. *Curr. Biol.* **12**, 1448–1461.

34. Robida-Stubbs S, Glover-Cutter K, Lamming DW, Mizunuma M, Narasimhan SD, Neumann-Haefelin E, Sabatini DM, Blackwell TK (2012) TOR signaling and rapamycin influence longevity by regulating SKN-1/Nrf and DAF-16/FoxO. *Cell Metab.* **15**, 713–724.

35. Dai Y, Zheng K, Clark J, Swerdlow RH, Pulst SM, Sutton JP, Shinobu LA, Simon DK (2014) Rapamycin drives selection against a pathogenic heteroplasmic mitochondrial DNA mutation. *Hum. Mol. Gen.* **23**, 637–647.

36. Li Q, Zhang T, Wang J, Zhang Z, Zhai Yu, Yang G, Sun X (2014) Rapamycin attenuates mitochondrial dysfunction via activation of mitophagy in experimental ischemic stroke. *Biochem. Biophys. Res. Commun.* **444**, 182–188.

37. Edinger AL, Linardic CM, Chiang G, Thompson CB, Abraham RT (2003) Differential effects of rapamycin on mammalian target of rapamycin signaling functions in mammalian cells. *Cancer Res.* **63**, 8451–8460.

38. Pivtoraiko VN, Harrington AJ, Mader BJ, Luker AM, Caldwell GA, Caldwell KA, Roth KA, Shacka J (2010) Low-dose bafilomycin attenuates neuronal cell death associated with autophagy-lysosome pathway dysfunction. *J. Neurochem.* **114**, 1193–1204.

39. Lode H (1991) The pharmacokinetics of azithromycin and their clinical significance. *Eur. J. Clin. Microbiol. Infect. Dis.* **10**, 807–812.

40. Cochemé HM, Murphy MP (2008) Complex I is the major site of mitochondrial superoxide production by paraquat. *J. Biol. Chem.* **283**, 1786–1798.

41. Park SK, Tedesco PM, Johnson TE (2009) Oxidative Stress and Longevity in *C. elegans* as Mediated by SKN-1. *Aging Cell*. **8**, 258–269.

42. Ray A, Martinez BA, Berkowitz LA, Caldwell GA, Caldwell KA (2014) Mitochondrial dysfunction, oxidative stress, and neurodegeneration elicited by a bacterial metabolite in a *C. elegans* Parkinson's model. *Cell Death Dis.* ***5***, e984.

43. Roberts TR, Dyson JS, Lane MCG (2002) Deactivation of the Biological Activity of Paraquat in the Soil Environment: a Review of Long-Term Environmental Fate. *J Agricult. Food Chem.* **50**, 3623–3631.

44. Lee SJ, Hwang AB, Kenyon C (2010) Inhibition of respiration extends *C. elegans*' lifespan via reactive oxygen species that increase HIF-1 activity. *Curr. Biol.* **20**, 2131–2136.

45. Yang W, Hekimi S (2010) A Mitochondrial Superoxide Signal Triggers Increased Longevity in Caenorhabditis elegans. *PLoS Biol.* **8**, e1000556.

46. Chang J, Rimando A, Pallas M, Camins A, Porquet D, Reeves J, Shukitt-Hale B, Smith MA, Joseph JA, Casadesus G (2012) Low-dose pterostilbene, but not resveratrol, is a potent neuromodulator in aging and Alzheimer's disease. *Neurobiol. Aging* **33**, 2062–2071.

47. Fischer N, Büchter C, Koch K, Albert S, Csuk R, Wätjen W (2017) The resveratrol derivatives trans-3,5-dimethoxy-4-fluoro-4′-hydroxystilbene and trans-2,4′,5-trihydroxystilbene decrease oxidative stress and prolong lifespan in Caenorhabditis elegans. *J. Pharm. Pharmacol.* **69**, 73–81.

48. Francioso A, Mastromarino P, Restignoli R, Boffi A, d'Erme M, Mosca L (2014) Improved stability of trans-resveratrol in aqueous solutions by carboxymethylated (1,3/1,6)-β-D-glucan. *J. Agricult. Food Chem.* **62**, 1520–1525.

49. Remsberg CM, Yáñez JA, Ohgami Y, Vega-Villa KR, Rimando AM, Davies NM (2008) Pharmacometrics of pterostilbene: preclinical pharmacokinetics and metabolism, anticancer, antiinflammatory, antioxidant and analgesic activity. *Phytother. Res.* **22**, 169–179.

50. Bertram R, Gram Pedersen M, Luciani DS, Sherman A (2006) A simplified model for mitochondrial ATP production. *J. Theo. Biol.* **243**(4), 575–586.

51. Saa A, Siqueira KM (2013) Modeling the ATP production in mitochondria. *Bull. Math. Biol.* **75**(9), 1636–1651.

52. Imai S, Guarente L (2014) NAD+ and sirtuins in aging and disease. *Trends Cell Biol.* **24**(8), 464–471.

53. Verdin E (2015) NAD^+ in aging, metabolism, and neurodegeneration. *Science* **350**(6265), 1208–1213.

54. Jasper H (2013) Sirtuins: Longevity focuses on NAD+. *Nat. Chem. Biol.* **9**(11), 666–667.

55. Blackwell TK, Steinbaugh MJ, Hourihan JM, Ewald CY, Isik M (2015) SKN-1/Nrf, stress responses, and aging in Caenorhabditis elegans. *Free Radic. Biol. Med.* **88**(B), 290–301.

56. Hesp K, Smant G, Kammenga JE (2015) Caenorhabditis elegans DAF-16/FOXO transcription factor and its mammalian homologs associate with age-related disease. *Exp. Gerontol.* **72**, 1–7.

57. Herndon LA, Schmeissner PJ, Dudaronek JM, Brown PA, Listner KM, Sakano Y, Paupard MC, Driscoll M (2002) Stochastic and genetic factors influence tissue-specific decline in ageing C. elegans. *Nature* **419**(6909), 808–814.

58. Hall DH, Gu G, García-Añoveros J, Gong L, Chalfie M, Driscoll M (1997) Neuropathology of degenerative cell death in Caenorhabditis elegans. *J. Neurosci.* **17**(3), 1033–1045.

59. Reina A, Subramaniam AB, Laromaine A, Samuel AD, Whitesides GM (2013) Shifts in the distribution of mass densities is a signature of caloric restriction in Caenorhabditis elegans. *PLoS ONE* **8**(7), e69651.
